# Molecular control of endurance training adaptation in male mouse skeletal muscle

**DOI:** 10.1038/s42255-023-00891-y

**Published:** 2023-09-11

**Authors:** Regula Furrer, Barbara Heim, Svenia Schmid, Sedat Dilbaz, Volkan Adak, Karl J. V. Nordström, Danilo Ritz, Stefan A. Steurer, Jörn Walter, Christoph Handschin

**Affiliations:** 1 Biozentrum, University of Basel, Basel, Switzerland; 2 Laboratory of EpiGenetics, Saarland University, Saarbrücken, Germany; 3 Present Address: University Hospital Basel, Basel, Switzerland; 4 Present Address: AstraZeneca, Mölndal, Sweden

**Keywords:** Metabolism, Transcriptomics, Proteomics, DNA methylation

## Abstract

Skeletal muscle has an enormous plastic potential to adapt to various external and internal perturbations. Although morphological changes in endurance-trained muscles are well described, the molecular underpinnings of training adaptation are poorly understood. We therefore aimed to elucidate the molecular signature of muscles of trained male mice and unravel the training status-dependent responses to an acute bout of exercise. Our results reveal that, even though at baseline an unexpectedly low number of genes define the trained muscle, training status substantially affects the transcriptional response to an acute challenge, both quantitatively and qualitatively, in part associated with epigenetic modifications. Finally, transiently activated factors such as the peroxisome proliferator-activated receptor-γ coactivator 1α are indispensable for normal training adaptation. Together, these results provide a molecular framework of the temporal and training status-dependent exercise response that underpins muscle plasticity in training.

## Abstract (web summary)

The authors explore the molecular signature of skeletal muscle adaptations to an acute bout of exercise in mice, providing a valuable resource that includes transcriptomic, epigenetic, proteomic and phosphoproteomic changes in muscle plasticity.

## Main

Skeletal muscle exerts pleiotropic functions, from thermoregulation to endocrine signalling by myokines and myometabolites, and detoxification of endogenous compounds, for example, kynurenines or aberrantly high levels of ketone bodies^1–5^. However, the main task of skeletal muscle is the generation of force for different types of contractile activities, including strength, endurance, fine motor control, posture and breathing. Skeletal muscle thus exhibits not only a broad morphological and functional specification, but also a remarkably adaptive plasticity to react to perturbations^4^. Remodelling of skeletal muscle requires interventions that disrupt homeostasis, to which muscle will progressively adapt only if repeated over time^3,4^. Morphologically, endurance training adaptations include mitochondrial expansion, vascularization and energy substrate storage^4^. In light of the powerful health benefits of exercise^6,7^, it is, however, surprising that the molecular underpinnings of muscle plasticity in exercise are still only rudimentarily understood^4^. In particular, the mechanistic framework that links the perturbations evoked by individual exercise bouts to long-term training adaptations are largely unknown^3,4^. In addition, it is unclear how the training status affects the molecular response to an acute bout of exercise and how changes in gene expression are ultimately linked to persistent modulation of protein levels, organelle function and tissue plasticity. The ‘repeated bout effect’ is based on the observation of reduced muscle damage and soreness in trained compared with untrained muscle^8,9^. Accordingly, a diminished amplitude in the expression of a number of genes in repeated exercise bouts has been reported, at least with a constant training load^10,11^, nevertheless presumably resulting in steady accumulation of transcripts, proteins and performance over time^12–17^. Such an encompassing model of transcriptional attenuation in training adaptation is, however, contradicted by different observations, for example, a broad-ranging qualitative and quantitative specification is implied by the vastly different epigenetic modifications in acute and chronic exercise settings^17–19^. Accordingly, the expression of many genes does not follow an attenuating pattern, but rather shows an exacerbated response in trained muscle, as described for the peroxisome proliferator-activated receptor-γ coactivator 1α (PGC-1α; gene symbol *Ppargc1a*)^20^. Collectively, little knowledge about the chronic, persistent mechanistic network in training adaptation exists.

To understand these fundamental aspects of muscle biology and plasticity, we therefore studied the acute maximal endurance exercise and chronic training response of mouse muscle in a systematic and comprehensive manner. Based on the interrogation of the molecular underpinnings of epigenetic, transcriptional, proteomic and phosphoproteomic changes, we provide a unique mechanistic framework of endurance training adaptations. The transcriptomic data of acute maximal exercise and chronic training are provided in the Myo-Transcriptome of Exercise database (Myo-TrEx: https://myo-trex.scicore.unibas.ch).

## Results

### A surprisingly low number of genes define the trained muscle

**Fig. 1:**
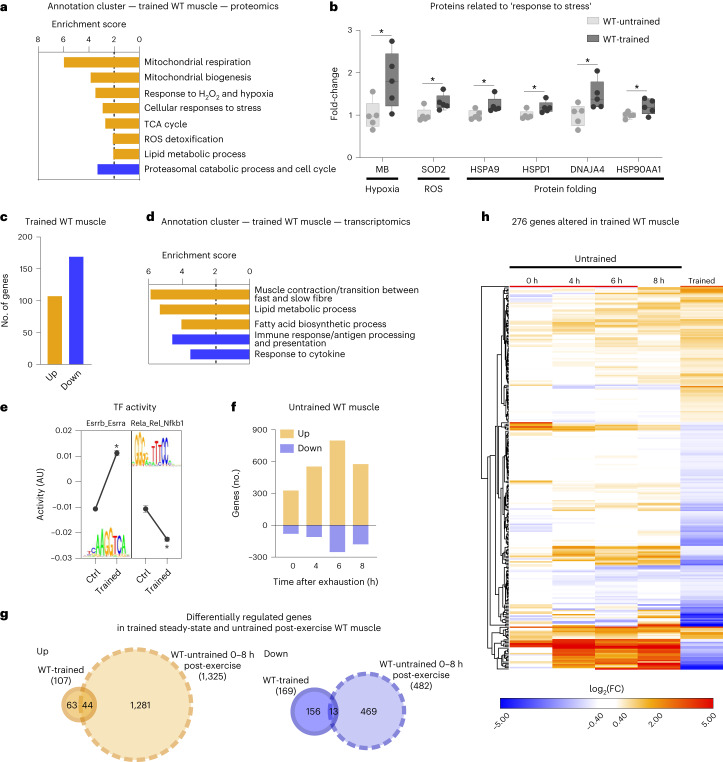
A low number of differentially expressed genes (DEGs) define a trained WT muscle. **a**, All functional annotation clusters of up- (orange) and downregulated (blue) proteins in trained muscle with an enrichment score >2. ROS, reactive oxygen species. **b**, Examples of proteins involved in the response to stress in sedentary untrained (light grey) and unperturbed trained (dark grey) muscle (box plots display the median and the 25th to 75th percentiles and whiskers indicate the minimal and maximal values). **c**, Number of genes differentially expressed in unperturbed trained muscle (cut-off: FDR < 0.05; log_2_(FC) ± 0.6). **d**, All functional annotation clusters of up- (orange) and downregulated (blue) genes in trained muscle with an enrichment score >2. **e**, Motifs of transcription factors from ISMARA that are among those with the highest and lowest activity. AU, arbitrary units. **f**, Number of genes after an acute bout of exhaustion exercise that are up- (orange) and downregulated (blue). **g**, Venn diagram of all genes that are changed in unperturbed trained muscle (orange is upregulated and blue downregulated) and those that are regulated after an acute bout of maximal exercise (light colour, dashed line). **h**, Heatmap of all genes differentially expressed in unperturbed trained muscle to visualize the overlap with acutely regulated genes using Euclidean distance hierarchical clustering for rows. The data are from five biological replicates and represent mean ± s.e.m. (if not otherwise indicated). Statistics of proteomics data were performed using empirical Bayes-moderated *t*-statistics as implemented in the R/Bioconductor limma package and for RNA-seq data with the CLC Genomics Workbench Software. Exact *P* values of proteomics data and *z*-scores of ISMARA data are displayed in Source data. The asterisk indicates difference to control (Ctrl; pre-exercise condition) if not otherwise indicated: in **b**, ^*^P < 0.05, in **e**, ^*^z-score > 1.96 (Extended Data Fig. 1 and Supplementary Tables 1–4). Source data

**Extended Data Fig. 1:**
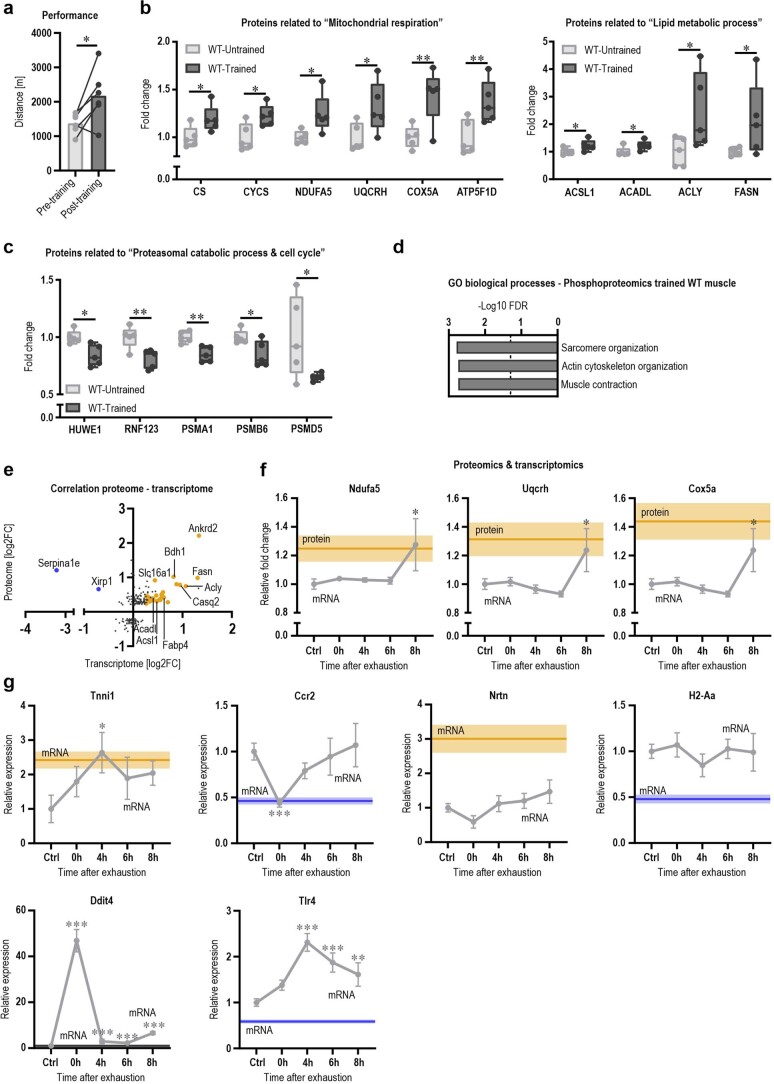
Proteomic and phosphoproteomic changes in a trained WT muscle. **a**, Changes in maximal running distance after 4 weeks of progressive treadmill training (n = 6 per group; mean difference (MD) = 802.3, 95% confidence interval (CI) = 80.62 to 1524, *p* = 0.0355). **b-c**, Examples of proteins represented in the annotations clusters of **b**) mitochondrial respiration and lipid metabolic process and **c**) proteasomal catabolic process in a sedentary untrained (light gray) and training (dark gray) muscle. **d**, All significant (FDR < 0.05) GO Biological Pathways of proteins with altered phosphorylation status after 4 weeks of training. **e**, Correlation plot of the transcriptome and proteome of trained muscle. Only depicting genes/proteins that are significantly altered in the proteomics analysis of a trained muscle (cutoff: *p* < 0.05; Log2FC ± 0.2). The colored (orange = up; blue = down) genes/proteins are also significantly altered on a transcriptional level in a trained muscle (FDR < 0.05; Log2FC ± 0.2). **f**, Representative proteins involved in mitochondrial respiration that are significantly increased in a trained muscle (solid orange line = mean ± SEM with light color) and acutely regulated on a transcriptional level post-exercise (gray line). **g**, The regulation of genes after one bout of maximal exercise in untrained muscle (gray line) as well as the steady-state level of the transcript in unperturbed trained muscle (solid line in orange = upregulated, blue = downregulated or gray = unchanged ± SEM in lighter color). Genes representing the different scenarios: same direction in both trained muscle as well as after an acute bout of maximal exercise; only changed in trained muscle; only regulated after an acute maximal exercise bout; or upregulated after an acute challenge and downregulated after training. Data from 5 biological replicates (if not otherwise indicated). Data represent means ± SEM (except for **b-c** where the box plots display the median and the 25^th^ to 75^th^ percentiles and whiskers indicate the minimal and maximal values). Statistics of proteomics data were performed using empirical Bayes moderated t-statistics as implemented in the R/Bioconductor limma package and for RNA-seq data with the CLC genomics workbench software. Exact *p*-values of proteomics data and FDR-values of RNA-seq data are displayed in the Source Data file. For the running distance (**a**) a paired two-tailed Student’s t-test was performed. * indicates difference to Ctrl (pre-exercise condition) if not otherwise indicated; *<0.05; **<0.01; ***<0.001. See also Fig. 1; Supplementary Tables 1, 2, 5. Source data

To study differences between untrained and endurance-trained muscles (in the present study, muscle always refers to quadriceps), mice were exercised by treadmill running on 5 d per week for 1 h. After 4 weeks, a significant improvement in running performance was observed (Extended Data Fig. 1a). A proteomic analysis also indicated a substantial remodelling of skeletal muscle (Fig. 1a and Supplementary Table 1). For example, proteins involved in mitochondrial respiration, lipid metabolism, oxygen transport or stress resilience are more abundant in trained than in untrained muscle (Fig. 1a,b, Extended Data Fig. 1b and Supplementary Table 2). In contrast, the levels of proteins linked to catabolic processes related to proteasomal degradation are mitigated by endurance training (Fig. 1a and Extended Data Fig. 1c), which, together with the induction of molecular chaperones, alludes to altered proteostasis. In contrast to the training-induced changes in protein abundance, training has very few effects on the steady-state phosphoproteome (differences in phosphorylation were observed in only 54 proteins). The corresponding proteins are mainly involved in cytoskeletal structure, sarcomere organization and muscle contraction, and are thus most probably linked to long-lasting alterations of contractility (Extended Data Fig. 1d and Supplementary Tables 1 and 2). According to prevailing models, the proteomic changes are brought about by a persistent modulation of gene expression with repeated exercise bouts^13^. To test this, we assessed the transcriptomic landscape of the trained muscle. Intriguingly, <2% of the detected genes were significantly changed in a trained muscle, with most transcripts being downregulated (Fig. 1c). Collectively, these genes define long-term cellular changes, for example, related to fibre-type switch, lipid metabolic processes or decreased inflammation (Fig. 1d and Supplementary Table 3). In line with these observations, Integrated System for Motif Activity Response Analysis (ISMARA)^21^ revealed a modulation of the predicted activity of only 22 transcription factors, for example, higher activity of the Esrrb_Esrra and lower activity of the Rela_Rel_Nfkb1 motifs (Fig. 1e and Supplementary Table 4). The genes that are altered in a trained muscle show only a small overlap with proteomic changes, suggesting that the proteome of a trained muscle is only to a small extent maintained transcriptionally. It is interesting that the subset of proteins with corresponding gene expression changes are predominantly involved in the lipid metabolic process (Extended Data Fig. 1e and Supplementary Table 5). Thus, most of the proteins that define the long-term plasticity of a trained muscle are not directly linked to a corresponding persistent transcriptional response.

These unexpected results raised the question of whether perturbations evoked by an acute maximal exercise bout activate transcriptional networks that encode the biological programmes observed in trained muscle. To test this hypothesis, untrained mice were exercised to exhaustion by treadmill running and the muscle transcriptome was assessed 0, 4, 6 and 8 h post-exhaustion. Similar to other studies, we found a large number of gene-regulatory events in this context of acute maximal exercise in untrained muscle, peaking 6 h post-exhaustion (Fig. 1f). A subset of these acute changes correlated with the accumulation of proteins in a trained muscle. These proteins were mostly upregulated and predominantly involved in mitochondrial respiration (Extended Data Fig. 1f and Supplementary Table 5). These genes are increased at the 8-h timepoint, suggesting that the induction of mitochondrial genes occurs several hours post-exercise and might even further increase at later timepoints. Intriguingly, the acutely regulated genes only poorly overlapped with persistent transcriptomic changes in trained muscle, because only 21% (57 of the 276 in total, with 44 of 107 up- and 13 of 169 downregulated) of the genes modulated in an unperturbed trained muscle are also regulated acutely in untrained muscle (Fig. 1g,h and Extended Data Fig. 1g). In fact, some of the genes exhibited the opposite regulation (Fig. 1h and Extended Data Fig. 1g), for example, reflected in transcripts related to inflammation (up acutely post-exercise, down in trained muscle).

### Acute response to exercise is training status dependent

**Fig. 2:**
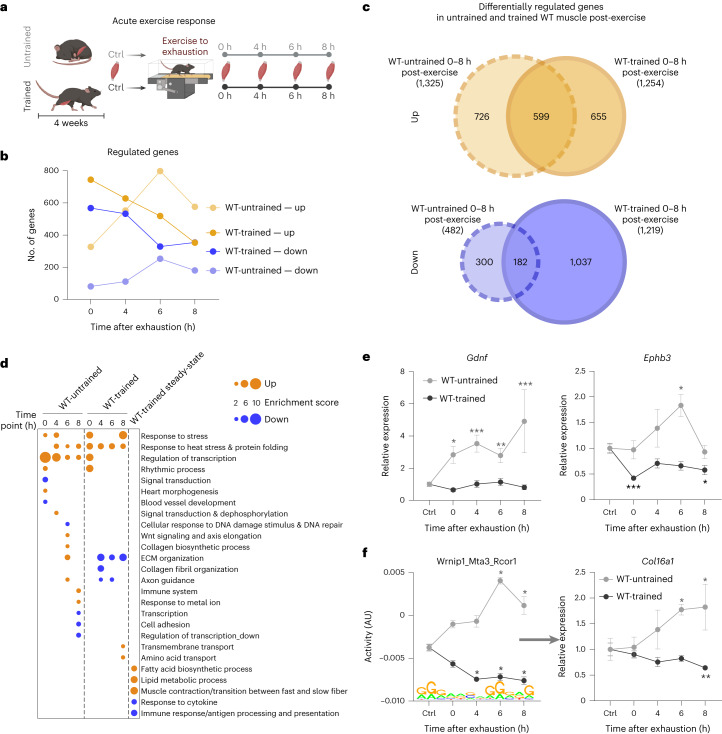
Qualitative transcriptional response to exercise depends on training status. **a**, Schematic representation of the experimental setup (illustration was created using BioRender.com with permission). **b**, Number of genes differentially expressed immediately (0 h), 4, 6 and 8 h after an acute bout of exhaustion exercise (cut-off: FDR < 0.05; log_2_(FC) ± 0.6) in untrained and trained muscle. **c**, Venn diagram of all significantly up- (orange) and downregulated (blue) genes (all timepoints merged) in untrained (light colour, dashed line) and trained (dark colour, solid line) muscle. **d**, Dot plot of all functional annotation clusters of up- (orange) and downregulated (blue) genes in untrained and trained muscle post-exercise, as well as unperturbed trained muscle with an enrichment score >2. **e**, Examples of gene trajectories in untrained (light grey) and trained (dark grey) muscle involved in axon guidance. **f**, Motif activities from ISMARA and expression changes of a predicted target gene that show an opposite regulation in untrained and trained muscle. The data are from five biological replicates and present mean ± s.e.m. Statistics of RNA-seq data were performed using the CLC Genomics Workbench Software. Exact FDR values of RNA-seq data and *z*-scores of ISMARA data are displayed in Source data. The asterisk indicates difference to Ctrl (pre-exercise condition): ^*^P < 0.05 (for motif activity: ^*^z-score > 1.96); ^**^P < 0.01; ^***^P < 0.001 (Extended Data Figs. 2–4 and Supplementary Tables 3 and 4). Source data

**Extended Data Fig. 2:**
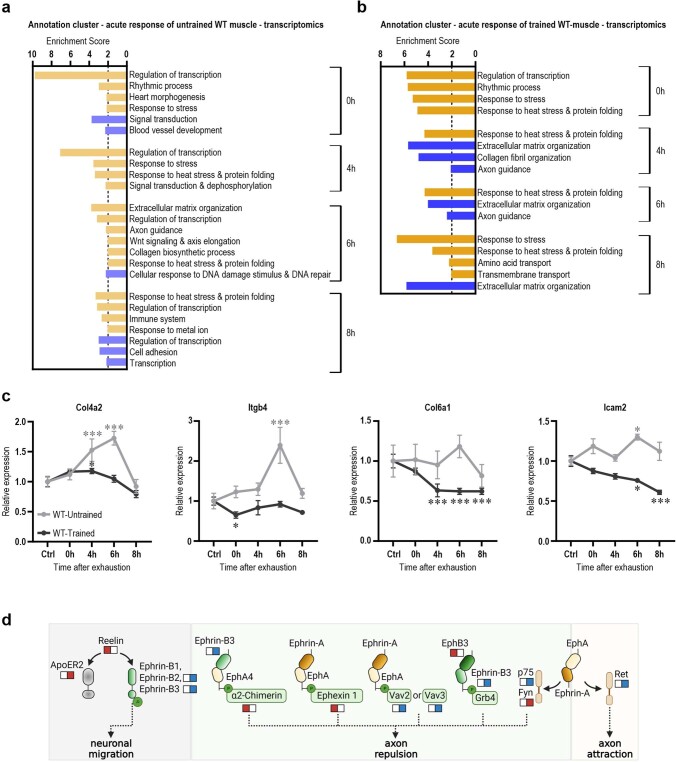
Distinct gene sets induced upon acute exercise in untrained and trained WT muscle. **a-b**, All functional annotation clusters of up- (orange) and downregulated (blue) genes after an acute maximal exercise bout in untrained (**a**) and trained (**b**) muscle with an enrichment score >2. **c**, Examples of gene trajectories in untrained (light gray) and trained (dark gray) muscle involved in ECM organization. Exact FDR-values are displayed in Source Data file. **d**, Schematic representation of genes involved in axon guidance and the possible functional consequences. The left square below each gene name represents the untrained response and the right square the trained response. Red = upregulated; blue = downregulated (illustration was created with BioRender.com with permission). Data from 5 biological replicates. Data represent means ± SEM. Statistics of RNA-seq data were performed with the CLC genomics workbench software. * indicates difference to Ctrl (pre-exercise condition); *<0.05; ***<0.001. See also Fig. 2; Supplementary Table 3. Source data

**Extended Data Fig. 3:**
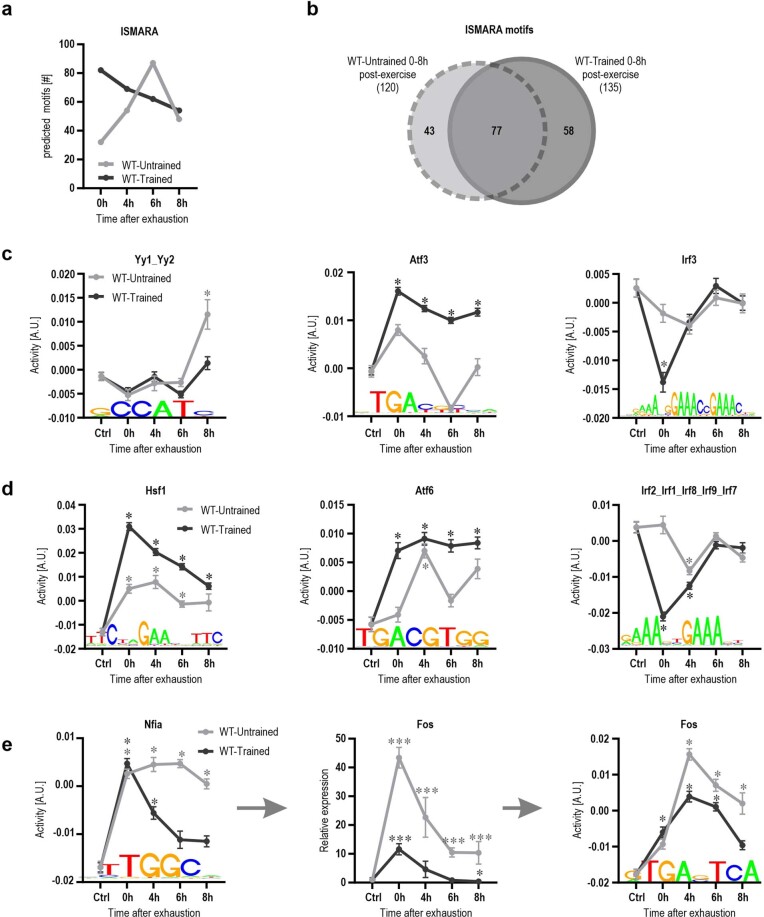
Divergent predicted motif activities of transcription factors in untrained and trained WT muscle. **a**, Number of motifs of transcription factors from ISMARA that have an increased or decreased activity (*z*-score >1.96) per time point in an untrained (light gray) and trained (dark gray) muscle after an acute maximal exercise bout. **b**, Venn diagram of all the predicted motifs that are changes in untrained (light gray) and trained (dark gray) muscle after an acute exercise bout. **c-d**, Trajectories of motif activity of transcription factors from ISMARA in untrained (light gray) and trained (dark gray) muscle post-exercise that are either specific to training status (**c**) or show an exacerbation after training (**d**). **e**, Example of a possible transcriptional cascade including a top predicted transcription factor by ISMARA and one of the downstream targets (gene expression and motif activities). Exact FDR-values of RNA-seq data and *z*-scores of ISMARA data are displayed in the Source Data file. Data from 5 biological replicates. Data represent means ± SEM. Statistics of RNA-seq data were performed with the CLC genomics workbench software. * indicates difference to Ctrl (pre-exercise condition); *<0.05 (for motif activity: * *z*-score >1.96); ***<0.001. See also Fig. 2; Supplementary Table 4. Source data

As the acute maximal exercise response in untrained muscle was not predictive of training adaptation, we next investigated the response of a trained muscle to an acute bout of maximal endurance exercise at the same four timepoints (Fig. 2a). Accordingly, mice that were trained for 4 weeks performed an exhaustive bout of treadmill running (Fig. 2a). Strikingly, the transcriptomic responses of untrained and trained muscle to an acute maximal endurance exercise bout were decisively different, qualitatively and quantitatively, the latter in terms of both amplitude (extent of change, that is, attenuated or exacerbated) and phase (temporal regulation, that is, induction of gene expression at different timepoints) (Fig. 2b,c). First, less than half of the upregulated genes overlapped between these two conditions and an even smaller proportion of the downregulated transcripts, of which a greater number were altered in the trained condition (Fig. 2c). Functionally, many of the acutely regulated genes in untrained muscle cluster with regulation of transcription and various aspects of stress response, damage, axon guidance and extracellular matrix (ECM) organization (Fig. 2d, Extended Data Fig. 2a and Supplementary Table 3). Strikingly, in regard to ECM remodelling and axon guidance, the functional prediction of the acute response of trained muscle was diametrically opposite to that of the untrained muscle (Fig. 2d,e, Extended Data Fig. 2a–d and Supplementary Table 3). ISMARA confirmed the substantial regulatory diversification (Extended Data Fig. 3a–e and Supplementary Table 4). Although approximately 35–43% of the motifs are specific to the training status (Extended Data Fig. 3b), many of the common motifs (*n* = 77) show altered trajectories and/or amplitudes (Extended Data Fig. 3c–e). In fact, 18 of the 77 motifs in the overlap significantly differed in amplitude. Moreover, in an additional 22 of the 77 motifs, the activity profiles point in the opposite direction. For example, the Wrnip1_Mta3_Rcor1 motif activity is higher in untrained and lower in trained muscle and, based on the association with collagen formation, could contribute to the distinct patterns of ECM remodeling (Fig. 2f). Thus, of the 178 predicted transcription factor motif activities after an acute bout of maximal exercise (in untrained and trained), only 21% (37 out of 178 motifs) exhibited a shared direction and amplitude, implying a strong regulatory diversification between these two conditions.

**Extended Data Fig. 4:**
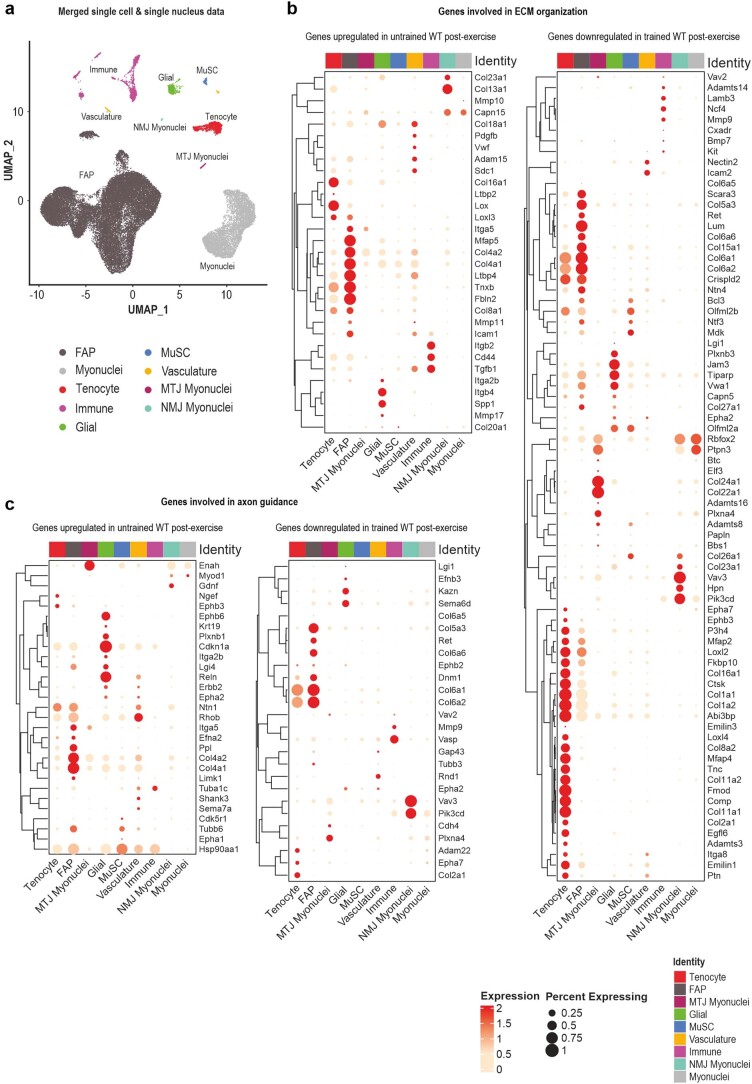
Cellular specification of the transcriptional response to exercise depends on training status. **a**, UMAP plot of public available single cell and single nucleus RNA-seq datasets^22,23^ to demonstrate the cellular specification of the exercise response in muscle (FAP = fibro-adipogenic progenitors; MuSC = muscle stem cells; MTJ = myotendinous junction; NMJ = neuromuscular junction). **b-c**, Deconvolution of genes involved in ECM remodeling (**b**) and axon guidance (**c**) that are upregulated in untrained and downregulated in trained muscle. See also Fig. 2. Source data

Many of the predicted functions, including a modulation of ECM remodeling, axon guidance and inflammation, could originate from non-myocytes in muscle tissue. Therefore, we performed cellular deconvolution of the bulk results with published single-cell RNA-sequencing (scRNA-seq) and single-nucleus RNA-sequencing (snRNA-seq) data of untrained muscle (Extended Data Fig. 4a)^22,23^. These analyses imply a surprisingly detailed specification of gene expression across different cell types (Extended Data Fig. 4b,c). For example, the cellular origin of ECM remodelling genes could mainly be fibroadipogenic progenitors in untrained muscle, complemented by tenocytes in trained muscle. This type of analysis is only predictive and cannot differentiate between changes in cell composition (that is, reduction in the number of tenocytes that could result in a relative downregulation of tenocyte-specific genes) or selective repression of the specific genes in stable populations (that is, lower number of transcripts per cell). However, regardless of the precise mechanism, such cell type-specific responses presumably result in the correspondingly distinct outcomes for ECM remodeling, axon guidance and potentially other functions after an acute endurance exercise bout in untrained compared with trained muscle. Future studies should therefore dissect the acute exercise and chronic training response of muscle tissue at the single-cell and single-nucleus levels.

**Fig. 3:**
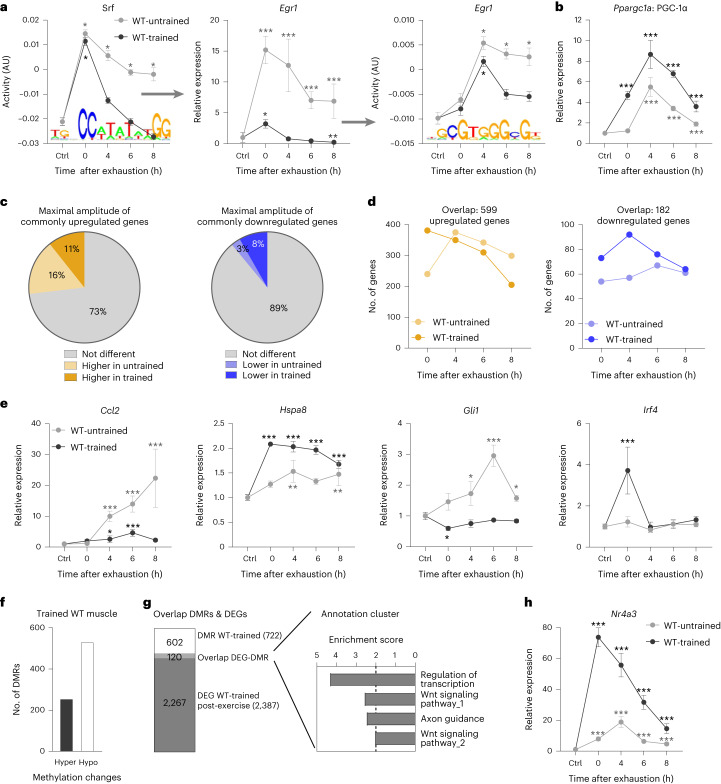
Faster transcriptional response in trained WT muscle after one bout of exhaustion exercise. **a**, Example of a possible transcriptional cascade including a top predicted transcription factor by ISMARA and one of the downstream targets (gene expression and motif activities). **b**, Example of a transcriptional regulator with distinct trajectories in untrained (light grey) and trained (dark grey) muscle. **c**, Proportion of commonly regulated genes with the same maximal amplitude (grey), higher amplitude in untrained muscle (light colour) or higher amplitude in trained muscle (dark colour). **d**, Visualization of the temporal trajectories of the commonly regulated genes (overlap from Fig. 2c) in untrained (light colour) and trained (dark colour) muscle (orange is upregulated and blue downregulated). **e**, Examples of different gene trajectories in untrained and trained muscle after an acute maximal exercise bout representing the different training status-specific transcriptional scenarios. **f**, Number of DMRs in an unperturbed trained muscle (hypermethylated is shown as a solid bar and hypomethylated as an open bar) compared with untrained sedentary WT muscle. **g**, Bar Venn diagram of DMRs of an unperturbed trained muscle (white) and DEGs after acute maximal exercise in trained muscle (dark grey) and the functional annotation clusters of the overlap (light grey, *n* = 120) with an enrichment score >2. **h**, Example of a transcription factor that is differentially methylated in trained muscle and more highly expressed after exercise in trained compared with untrained muscle. The data are from five biological replicates and represent mean ± s.e.m. Statistics of RNA-seq data were performed using the CLC Genomics Workbench Software. Exact FDR values of RNA-seq data and *z*-scores of ISMARA data are displayed in Source data. Differences in relative expression changes presented in **d** were calculated using a two-tailed Student’s *t*-test. The asterisk indicates difference to Ctrl (pre-exercise condition): ^*^P < 0.05 (for motif activity: ^*^z-score > 1.96); ^**^P < 0.01; ^***^P < 0.001 (Extended Data Fig. 5 and Supplementary Tables 4, 6 and 7). Source data

**Extended Data Fig. 5:**
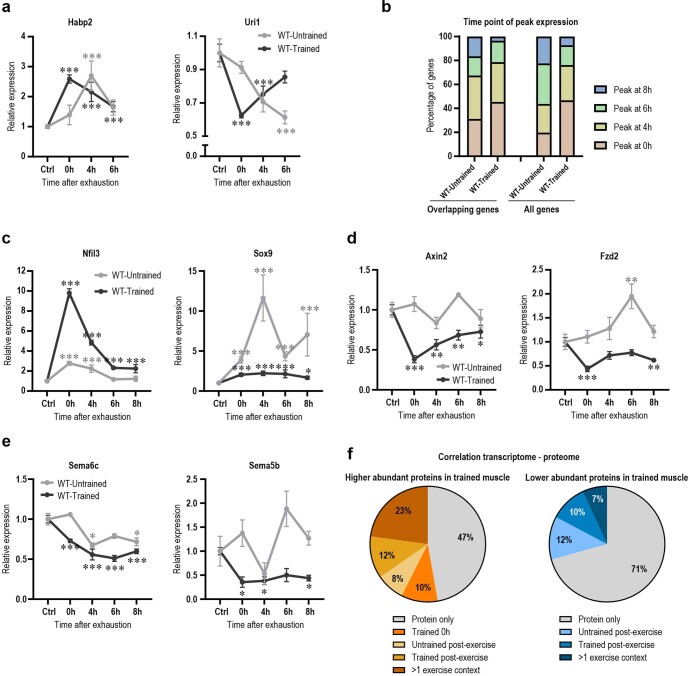
Quantitative differences between the exercise response of untrained and trained WT muscle. **a**, Representative examples of genes with the same maximal amplitude of gene expression (up- or downregulated) that show a phase shift towards an earlier time point in trained muscle after acute maximal exercise (dark gray) compared to untrained muscle (light gray). **b**, Representation of the time points when peak expression is reached in either the 599 commonly upregulated genes or all upregulated genes in untrained and trained muscle post-exercise. **c-e**, Examples of differentially methylated genes involved in transcription (**c**), Wnt signaling (**d**) and axon guidance (**e**) in untrained (light gray) and trained (dark gray) muscle post-exercise. **f**, Pie chart of the proteome of a trained muscle with the corresponding correlation with the transcriptome of an unperturbed trained muscle or an untrained or trained muscle after an acute bout of maximal exercise. The gray area depicts the proportion of proteins that are not regulated on a transcriptional level. The colored parts (orange = upregulated and blue = downregulated) represent an overlap with the transcriptome of either unperturbed trained muscle or untrained or trained muscle post-exercise. Data from 5 biological replicates. Data represent means ± SEM. Statistics of proteomics data were performed using empirical Bayes moderated t-statistics as implemented in the R/Bioconductor limma package and for RNA-seq data with the CLC genomics workbench software. All exact FDR-values are displayed in Source Data file. * indicates difference to Ctrl (pre-exercise condition); *<0.05; **<0.01; ***<0.001. See also Fig. 3; Supplementary Table 1. Source data

Besides qualitative differences in functional gene clusters between untrained and trained muscle, quantitative specification was observed for some common processes induced on an acute perturbation, for example, the regulation of transcription or the response to heat stress in a training status-dependent manner (Fig. 2d). For example, the modulation of the regulatory axis serum response factor–early growth response 1 indicates mitigation of the immediate early stress response in trained muscle (Fig. 3a and Extended Data Fig. 3e). Inversely, the expression of other transcriptional regulators such as PGC-1α is exacerbated, highlighting the specificity of gene-regulatory events in untrained and trained muscle (Fig. 3b). Intriguingly, contradicting the suggested attenuated response of a trained muscle^3,10,11,13–15^, the maximal amplitude of peak expression of most commonly regulated genes is very similar in untrained and trained muscle, specifically 73% of the shared upregulated and almost 90% of the shared downregulated genes (Fig. 3c and Extended Data Fig. 5a). However, a marked shift in the temporal trajectories was observed. For example, a substantially higher number of commonly regulated genes are already elevated at 0 h in trained muscle (Fig. 3d and Extended Data Fig. 5a). Furthermore, peak expression is also shifted towards the 0 h timepoint in all, as well as just the subset of the commonly regulated genes (Extended Data Fig. 5a,b). In fact, almost half of all upregulated genes in trained muscle peak at 0 h, whereas this applies to only ~20% of the upregulated genes in untrained muscle, where most peak after 6 h (Extended Data Fig. 5b). Overall, as opposed to the model of general attenuation of gene expression with training habituation^3,10,11,13–15^, our results suggest a much more complex picture, with noteworthy occurrence of all scenarios: attenuation, exacerbation and selective expression changes in untrained or trained muscle after an acute maximal exercise bout and, probably as important, a temporal shift in gene expression (Fig. 3e).

### Priming of regulatory genes by DNA methylation changes

The divergent specification and more rapid induction of gene expression in trained muscle suggest a priming to react to recurrent perturbations. Epigenetic changes, which can induce such a poised state, have been reported in training adaptation^17,19^. To test this, we performed reduced representation bisulphite sequencing (RRBS) to catalogue DNA methylation events in trained muscle (Fig. 3f and Supplementary Table 6). A very low number of differentially methylated regions (DMRs) are associated with gene expression changes in unperturbed trained muscle (*n* = 9). Intriguingly, a subset of these DMRs are in the immediate genomic vicinity of a group of genes (120 out of 2,387) that are regulated after an acute maximal exercise bout in trained muscle (Fig. 3g). These epigenetic modulations thus might not be associated with the persistent gene expression pattern in unperturbed trained muscle, but are more likely to contribute to a priming of transcriptional regulation to an acute bout of exercise. It is interesting that these genes enrich in functions related to the regulation of transcription, Wnt signalling and axon guidance signalling effectors (Fig. 3g,h, Extended Data Fig. 5c–e and Supplementary Table 7). For example, the induction of nuclear receptor 4A3 (*Nr4a3*), which is associated with DMRs in trained muscle, is not only greatly exacerbated in trained compared with untrained muscle, but also displays a phase shift towards a peak immediately post-exercise (Fig. 3h). Thus, epigenetic modifications could contribute to the different gene expression of a trained muscle to an acute perturbation, primarily affecting regulatory genes, with subsequent downstream consequences independent of DNA methylation changes.

Overall, very distinct transcriptomes were found in unperturbed trained, acutely exercised untrained and acutely exercise trained muscle. Although the correlation between the persistent proteomic and transcriptome changes in trained muscle is low, better results are achieved when integrating all gene expression changes, including those observed in acutely exercised untrained and trained animals. Collectively, these transcriptomic events correlate with the trained proteome up to 53% for upregulated and 30% for downregulated proteins for which transcript data are available (Extended Data Fig. 5f), highlighting the importance of broad comparisons between transcriptomes and proteomes^24^. So far, it is unclear whether the rest of the trained proteome (47% of upregulated proteins, which, for example, cluster in the tricarboxylic acid (TCA) cycle (Supplementary Table 5), and 70% of downregulated proteins) is evoked by post-translational processes, or linked to transcriptional events at other timepoints post-exhaustion and/or intermediate exercise bouts not included in the present study. Overall, these findings allude to a complex regulatory network, including transient and persistent transcriptional as well as post-translational events, mediating long-term proteomic adaptations.

### PGC-1α is indispensable for normal training adaptation

**Fig. 4:**
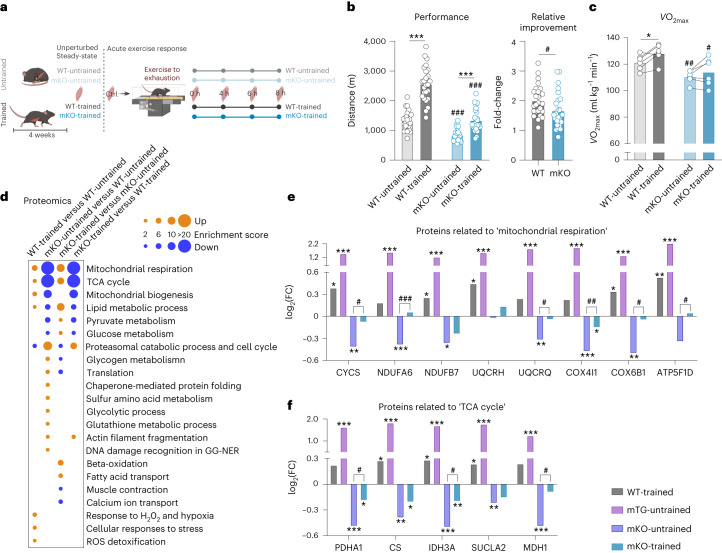
PGC-1α is indispensable for normal physiological responses to long-term training. **a**, Schematic representation of the experimental setup (illustration was created using BioRender.com with permission). **b**, Performance of untrained (light colour) and trained (dark colour) WT (grey) and mKO (blue) animals (WT-trained versus WT-untrained: mean difference (MD) = 1,242, 95% confidence interval (CI) = 946.1–1,539, *P* < 0.0001; mKO-trained versus mKO-untrained: MD = 500.9, 95% CI = 204.5–797.3, *P* = 0.0002; mKO-untrained versus WT-untrained: MD = −478.9, 95% CI = −775.3 to −182.5, *P* = 0.0003; and mKO-trained versus WT-trained: MD = −1,220, 95% CI = −1,517 to −924.1, *P* < 0.0001) and relative improvement of WT and mKO animals after 4 weeks of progressive treadmill training (MD = −0.3368, 95% CI = −0.6574 to −0.01625, *P* = 0.0399) (*n* = 25 biological replicates per group). **c**, Changes in *V*O_2max_ before (light colour) and after (dark colour) training (WT post-training versus WT pre-training: MD = 6.833, 95% CI = 0.5067–13.16, *P* = 0.350; mKO post-training versus mKO pre-training: MD = 3.667, 95% CI = −2.66 to 9.993, *P* = 0.2926; mKO-untrained versus WT-untrained: MD = −11.00, 95% CI = −17.87 to −4.132, *P* = 0.0051; and mKO-trained versus WT-trained: MD = −14.17, 95% CI = −25.67 to −2.659, *P* = 0.0207) (*n* = 6 biological replicates per group). **d**, Dot plot of all functional annotation clusters of significantly altered proteins with an enrichment score >2. **e**,**f**, Examples of proteins involved in mitochondrial respiration (**e**) and TCA cycle (**f**) in WT-trained (grey; *n* = 5), mTG-untrained (pink; *n* = 5), mKO-untrained (dark blue; *n* = 6) and mKO-trained (blue; *n* = 5). Values are expressed relative to untrained WT sedentary controls (*n* = 5). Statistics of proteomics data were performed using empirical Bayes-moderated *t*-statistics as implemented in the R/Bioconductor limma package. Exact *P* values are displayed in Source data. To assess differences between untrained and trained animals and between genotypes, two-way ANOVA followed by Šídák’s multiple-comparison test (**b** and **c**) or two-tailed Student’s *t*-test was performed (relative improvement in **b** and **c**). The asterisk indicates difference to Ctrl (pre-exercise condition) if not otherwise indicated; hashtag indicates differences to the same condition in WTs: ^*/#^*P* < 0.05; ^**/##^*P* < 0.01; ^***/###^*P* < 0.001 (Extended Data Fig. 6 and Supplementary Tables 1 and 2). Source data

**Extended Data Fig. 6:**
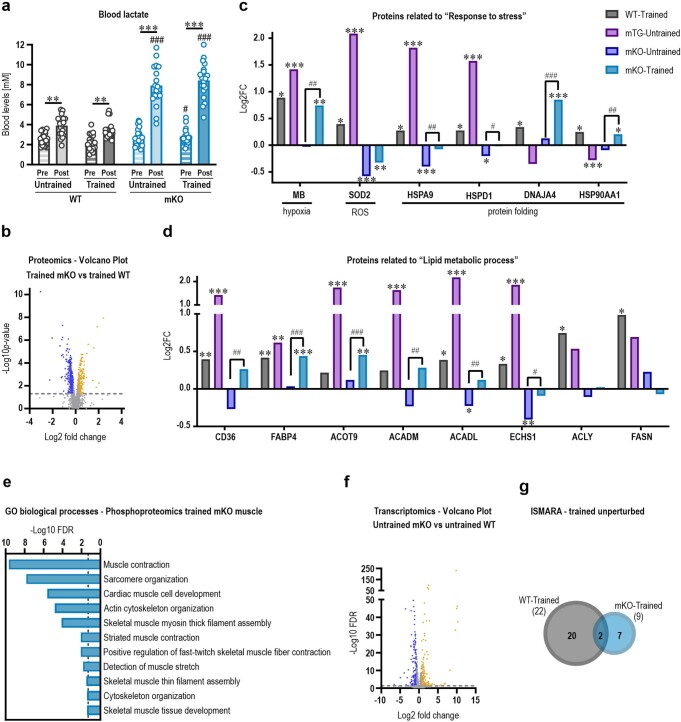
PGC-1α is essential for normal training adaptation. **a**, Blood lactate level pre- (bar with stripes) and post-exercise (filled bar) in untrained and trained WT (gray) and mKO (blue) animals after an acute bout of maximal exercise (WT-Untrained post vs WT-Untrained pre: MD = 1.332, 95% CI = 0.3739 to 2.289, *p* = 0.0027; WT-Trained post vs WT-Trained pre: MD = 1.2, 95% CI = 0.2666 to 2.133, *p* = 0.0063; mKO-Untrained post vs mKO-Untrained pre: MD = 5.145, 95% CI = 4.212 to 6.078, *p* < 0.0001; mKO-Trained post vs mKO-Trained pre: MD = 5.660, 95% CI = 4.727 to 6.593, *p* < 0.0001; mKO-Untrained pre vs WT-Untrained pre: MD = 0.2016, 95% CI = −0.3026 to 0.7058, *p* = 0.4231; mKO-Untrained post vs WT-Untrained post: MD = 4.015, 95% CI = 2.977 to 5.053, *p* < 0.0001; mKO-Trained pre vs WT-Trained pre: MD = 0.5450, 95% CI = 0.0586 to 1.031, *p* = 0.291; mKO-Trained post vs WT-Trained post: MD = 5.005, 95% CI = 4.122 to 5.888, *p* < 0.0001); n = 20 biological replicates per group. **b**, Volcano plot of the proteome of trained mKO muscle compared to trained WT muscle. **c-d**, Examples of proteins involved in the response to stress (**c**) and lipid metabolic process (**d**) in trained WT (gray; n = 5), untrained sedentary mTG (pink; n = 5), untrained sedentary mKO (dark blue; n = 6) and trained mKO (blue; n = 5). Values are expressed relative to the untrained sedentary WT control (n = 5). Exact *p*-values are displayed in Supplementary Table 1. **e**, All significant (FDR < 0.05) GO Biological Pathways of proteins with altered phosphorylation status after 4 weeks of training in mKO (compared to untrained sedentary mKO). **f**, Volcano plot of the transcriptome of untrained sedentary mKO muscle compared to that of untrained sedentary WT muscle. **g**, Venn diagram of all predicted motifs that are changes in unperturbed trained WT (gray) and mKO (blue) muscle. Data from 5 biological replicates (if not otherwise indicated). Statistics of proteomics data were performed using empirical Bayes moderated t-statistics as implemented in the R/Bioconductor limma package and for RNA-seq data with the CLC genomics workbench software. Exact *p*-values of proteomics data and FDR-values of RNA-seq data are displayed in the Source Data file. To assess differences between untrained and trained animals as well as between genotypes in panel **a**, two-way ANOVA followed by Šídák’s multiple comparisons test (repeated measures pre-post) or two-tailed Student t-test were performed (between genotypes of the same condition) * indicates difference to Ctrl (pre-exercise condition in **a** or untrained sedentary WT in **c-d**); # indicates difference between mKO and WT of the same condition in panel **a** or between mKO-Trained and mKO-Untrained in panels **c-d**; *<0.05; **<0.01; ***<0.001. See also Fig. 4; Supplementary Tables 1, 2, 4. Source data

Notably, many transcriptional regulators that are engaged strongly and early after acute maximal exercise exhibit a diversification between the first bout (in an untrained muscle) and after a period of training, including PGC-1α (Fig. 3b). This coregulator protein has been implicated in the acute response by integrating various signalling pathways and subsequently affecting the activity of numerous transcription factors, thereby controlling a complex transcriptional network^25^. Our observation, recapitulating previous results in human muscle^20^, of a quantitative difference of PGC-1α on exercise in trained compared with untrained muscle, would indicate that PGC-1α not only controls an acute stress response, but also might affect the transcriptome of exercised muscle in the trained state. Nevertheless, gene expression changes of this regulatory nexus are only transient and not preserved in unperturbed trained muscle. Thus, the relevance of adequate regulation and function of PGC-1α in long-term training adaptations has been questioned and, at least in part, conflicting findings have been reported^26–29^. To obtain comprehensive information on muscle PGC-1α in training, we therefore repeated the exercise study with muscle-specific PGC-1α knockout (mKO) mice (Fig. 4a). In agreement with previous work^30^, mKO mice exhibit a reduced endurance capacity, running approximately 40% less than wild-type (WT) controls (Fig. 4b). Despite these limitations, the PGC-1α loss-of-function animals substantially improved maximal performance after 4 weeks of training, in relative and absolute terms, reaching the levels of untrained WT mice, thus still significantly less than the trained WT counterparts (Fig. 4b). Importantly, blood lactate levels post-exercise were higher in mKO compared with WT animals, which implies a higher reliance on anaerobic processes to generate ATP (Extended Data Fig. 6a). Moreover, maximal oxygen consumption (*V*O_2max_) failed to improve in mKOs (Fig. 4c), alluding to an alternative adaptation of endurance capacity in these mice. Such an abnormal endurance training adaptation was substantiated by the proteomic analysis of trained muscle of WT and mKO mice (Extended Data Fig. 6b and Supplementary Table 1). First, many of the training-regulated proteins involved in mitochondrial respiration, the lipid metabolic process and the TCA cycle are already found at lower levels in sedentary mKO compared with sedentary WT animals (Fig. 4d–f, Extended Data Fig. 6c,d and Supplementary Tables 1 and 2). Although many of these proteins can be modulated in mKO mice by training, most do not even reach levels normally seen in sedentary WT muscle. Similar to trained WT muscle, relatively few proteins show altered phosphorylation levels (103 proteins; Supplementary Table 1). These proteins are predominantly involved in sarcomere organization and muscle contraction (Extended Data Fig. 6e and Supplementary Table 2).

**Fig. 5:**
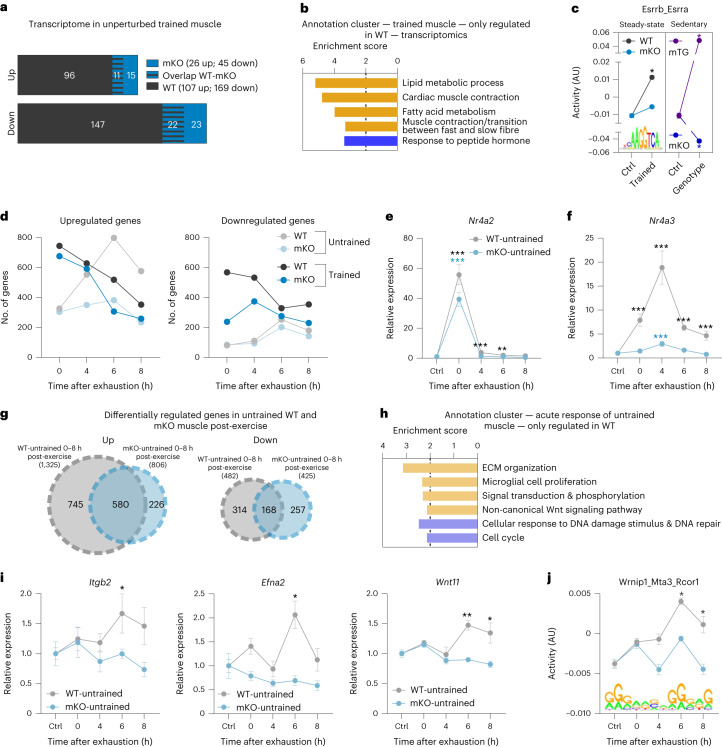
PGC-1α is indispensable for the normal transcriptional response to acute maximal exercise. **a**, Bar Venn diagram of the genes altered in unperturbed trained WT (grey) and mKO (blue) muscle. **b**, All functional annotation clusters of genes that are only up- (orange) and downregulated (blue) in trained muscle of WT animals (up: *n* = 96; down: *n* = 147) with an enrichment score >2. **c**, Motif of the transcription factors from ISMARA with the most significant activity change in trained WT animals and the comparison of the activity in trained mKO muscle (left blue), gain-of-function model (sedentary muscle-specific PGC-1α transgenics (mTG), purple) and loss-of-function model (sedentary mKO, dark blue). **d**, Number of genes that are up- and downregulated 0, 4, 6 and 8 h after an acute maximal exercise bout in untrained WT (light grey), trained WT (dark grey), untrained mKO (light blue) and trained mKO (dark blue) animals. **e**,**f**, Examples of gene trajectories with the peak expression immediately post-exercise (**e**) or at a later time (**f**) in untrained WT and mKO animals. **g**, Venn diagrams of all up- and downregulated genes after an acute bout of exercise in untrained WT (light grey) and mKO (light blue) mice. **h**, All functional annotation clusters of up- (orange) and downregulated (blue) genes that are regulated only in untrained WT mice (745 genes up- and 314 genes downregulated) with an enrichment score >2. **i**, Examples of genes involved in ECM organization, microglial cell proliferation and Wnt signalling that are regulated only in WT muscle. **j**, Prediction of the activity of a motif using ISMARA that is changed only in WT muscle and might be involved in the regulation of ECM-related genes. The data are from five biological replicates and represent mean ± s.e.m (if not otherwise stated). Statistics of RNA-seq data were performed using the CLC Genomics Workbench Software. Exact FDR values of RNA-seq data and *z*-scores of ISMARA data are displayed in Source data. The asterisk indicates difference to Ctrl (pre-exercise condition): ^*^P < 0.05 (for motif activity: ^*^z-score > 1.96); ^**^P < 0.01; ^***^P < 0.001 (Extended Data Fig. 7 and Supplementary Tables 4 and 8). Source data

Next, we investigated how the altered phenotypic and proteomic adaptations of trained muscles lacking PGC-1α are reflected in the transcriptome. In sedentary mice, the lack of PGC-1α causes a pronounced transcriptional suppression of genes involved in the lipid metabolic process (Extended Data Fig. 6f and Supplementary Table 8). Then, even when compared with the already constrained transcriptional changes in unperturbed trained WT muscle, far fewer genes were regulated by training in the absence of PGC-1α (Fig. 5a). Of note, 90% of all upregulated and 87% of all downregulated, transcriptional events were dependent on the presence of PGC-1α in WT muscle (Fig. 5a). Many of these genes encode proteins of lipid metabolism and the fast-to-slow muscle fibre transition (Fig. 5b and Supplementary Table 8). Even more impressive, in the unperturbed trained muscle, almost all (91%) transcription factor motif activities were affected by the loss of function of PGC-1α (Extended Data Fig. 6g and Supplementary Table 4). Most notably, ISMARA analysis revealed that the significant training-linked increase in Esrrb_Esrra motif activity, a binding site for the oestrogen-related receptor-α, was completely blunted in mKO mice (Fig. 5c). In line with this, the activity of this motif was highly increased in muscle-specific PGC-1α gain-of-function transgenic mice (mTG) (Fig. 5c). The phenotypic, proteomic and transcriptomic data thus strongly indicate that PGC-1α is indispensable for a normal, physiological training response, even though this factor is only transiently engaged in acute exercise bouts.

### Normal transcriptional exercise response depends on PGC-1α

**Extended Data Fig. 7:**
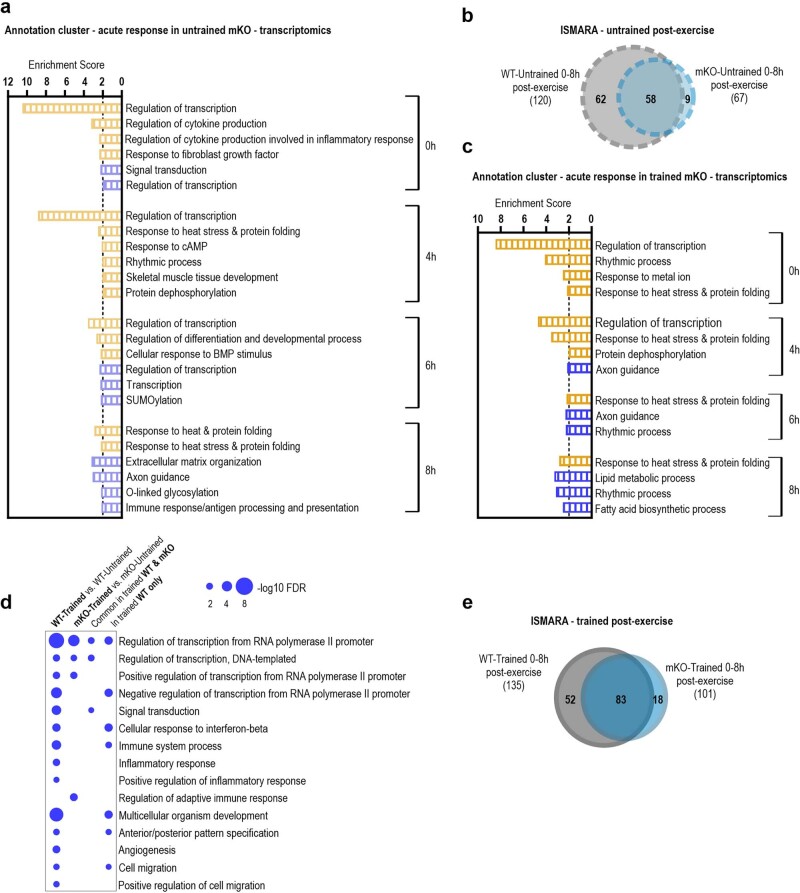
Altered transcriptional response to acute exercise in muscles lacking PGC-1α. **a**, All annotation clusters of genes that are significantly up- (orange) or downregulated (blue) in untrained mKO animals after a maximal exercise bout with an enrichment score >2. **b**, Venn diagram of all predicted motifs that are changes after an acute maximal exercise bout in untrained WT (gray) and mKO (blue) animals. All time points are merged. **c**, All annotation clusters of genes that are significantly up- (orange) or downregulated (blue) in trained mKO animals after a maximal exercise bout with an enrichment score >2. **d**, Dot plot of all significant (FDR < 0.05) GO Biological Processes of the downregulated genes at the 0 h time point in trained WT and mKO animals. The two left columns represent all downregulated genes at 0 h in trained WT compared to untrained WT and trained mKO compared to untrained mKO. The third column represents genes that are downregulated in both the trained WT and mKO compared to the respective control. The last column represents the genes that are only downregulated at the 0 h time point in the trained WT animals, but not the mKOs. **e**, Venn diagram of all predicted motifs that are changes after an acute exercise bout in trained WT (gray) and mKO (blue) animals. All time points are merged. Data from 5 biological replicates. Statistics of RNA-seq data were performed with the CLC genomics workbench software. See also Figs. 5–6; Supplementary Tables 4, 8. Source data

Next, we assessed whether the marked differences in the long-term adaptation to training in muscles lacking PGC-1α are reflected in the response to acute maximal exercise (Fig. 4a). First, immediately post-exercise (0 h), the response of WT and mKO animals is relatively similar in terms of the number of DEGs as well as the amplitude of the gene expression (Fig. 5d,e). However, in untrained mKO muscle, a massive blunting of transcriptional induction at the later timepoints (4–8 h post-exercise) was found, so subsequent to the physiological PGC-1α elevation in WT muscle (Fig. 5d,f). Taken together, substantial qualitative differences in gene expression emerged, with 56% (745 out of 1,325) of all upregulated, and 65% (314 out of 482) of all downregulated genes being dependent on the presence of PGC-1α (Fig. 5g). Functional annotation revealed that many of these genes encode proteins involved in ECM organization, signal transduction, cell cycle/proliferation and other processes (Fig. 5h,i). In untrained mKO muscle, the transcriptomic response to acute maximal exercise was characterized by a modulation of genes related to inflammation and an inverse regulation of genes involved in axon guidance (up in WT, down in mKO) (Extended Data Fig. 7a). Finally, the divergent transcriptomic response was linked to a substantial regulatory rewiring: 52% (62 out of 120) predicted motif activities associated with the acute maximal exercise response of untrained WT muscle were lost in mKOs (for example, Wrnip1_Mta3_Rco1, linked to ECM remodelling) (Fig. 5j, Extended Data Fig. 7b and Supplementary Table 4).

**Fig. 6:**
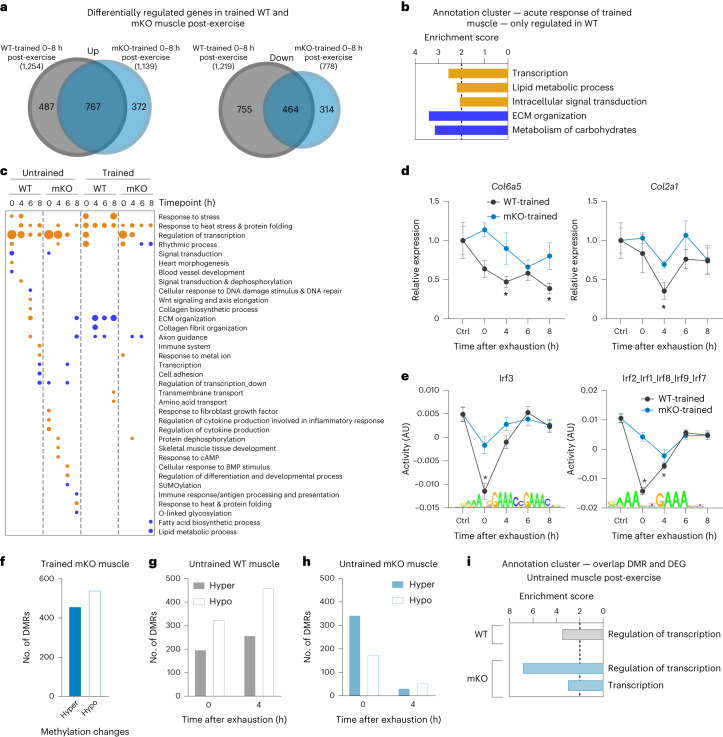
PGC-1α controls exercise-linked DNA methylation events. **a**, Venn diagrams of all up- and downregulated genes after an acute bout of maximal exercise in trained WT (dark grey) and mKO (dark blue) mice. **b**, All functional annotation clusters of up- (orange) and downregulated (blue) genes that are regulated only in trained WT mice (487 genes up- and 755 genes downregulated) with an enrichment score >2. **c**, Dot plot of all functional annotations clusters of up- (orange) and downregulated (blue) genes after an acute bout of maximal exercise in untrained and trained WT and mKO animals. **d**, Examples of genes involved in ECM organization in trained WT (grey) and mKO (blue) mice. **e**, Prediction of the activity of motifs using ISMARA that are changed only in WT muscle and linked to inflammation. **f**, Number of DMRs in trained mKO compared with untrained mKO muscle (hypermethylated is shown as a solid bar and hypomethylated as an open bar). **g**,**h**, Number of hyper- (solid bars) and hypomethylated (open bars) regions 0 and 4 h after exhaustion in untrained WT (**g**) and untrained mKO (**h**) animals compared with untrained sedentary animals of the respective genotype. **i**, All functional annotation clusters of genes that are differentially methylated and transcriptionally regulated after an acute bout of exercise in untrained WT (grey) and mKO (blue) mice. The data are from five biological replicates. Statistics of RNA-seq data were performed using the CLC Genomics Workbench Software. Exact FDR values of RNA-seq data and *z*-scores of ISMARA data are displayed in Source data. The asterisk indicates difference to control animals of the respective genotype: ^*^P < 0.05 (for motif activity: ^*^z-score > 1.96). (Extended Data Figs. 7 and 8 and Supplementary Tables 3, 4 and 6–8). Source data

Next, we compared the acute exercise response of trained mKO muscle with trained WT muscle. First, the temporal shift of gene expression towards 0 h was observed in both WT and mKO muscles (Fig. 5d). Second, even though only 39% (487 out of 1,254) of the upregulated genes were PGC-1α dependent in trained muscle, the proportion of commonly PGC-1α-dependent, downregulated genes (62%, 755 out of 1,219) remained similar to that found in untrained muscle (Fig. 6a). Functionally, these genes encode proteins involved in transcription, and metabolism of lipids and carbohydrates, as well as ECM remodelling (Fig. 6b and Supplementary Table 8). Intriguingly, the increase and decrease in ECM remodelling in acute maximal exercise of untrained and trained WT muscles, respectively, both seem to be dependent on the presence of this coregulator (Figs. 5h and 6b–d and Extended Data Fig. 7a,c). Of note, there is a prominent difference in the number of downregulated genes immediately post-exercise (0 h) between WT and mKO muscles (Fig. 5d). The genes that are reduced only in acutely exercised, trained WT muscle, and not in the corresponding mKO counterpart, were associated with inflammation (Extended Data Fig. 7d and Supplementary Table 8), in line with the higher activity-dependent muscle damage and inflammation that have previously been reported in mKO muscles^30^. In the trained muscle, acute exercise exhibited 39% (52 out of 135) of predicted transcription factor activities to be absent in the mKO muscles, for example, that of Irf3 and Irf2_Irf1_Irf8_Irf9_Irf7, regulating inflammation-related genes (Fig. 6e, Extended Data Fig. 7e and Supplementary Table 4).

### PGC-1α controls exercise-linked DNA methylation events

**Extended Data Fig. 8:**
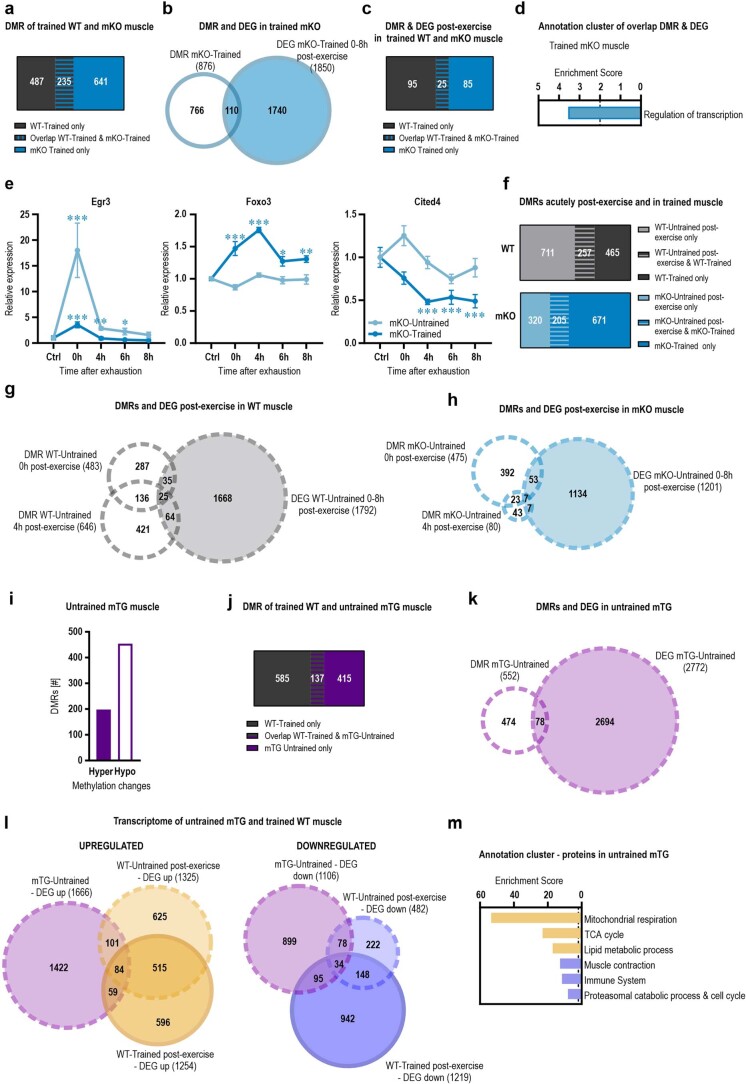
Gain- and loss-of-function of PGC-1α affects DNA methylation events. **a**, Bar Venn diagram of differentially methylated regions (DMRs) of an unperturbed trained WT (dark gray) and mKO (dark blue) muscle. The common DMR are striped (gray and blue). **b**, Venn diagram of DMRs of an unperturbed trained mKO muscle (open circle) and differentially expressed genes (DEG) after acute maximal exercise in trained mKO muscle (blue circle). **c**, Bar Venn diagram of the DMRs that are associated with acute gene expression changes (overlap Fig. 3g and panel b of this figure) of WT (dark gray) and mKO (dark blue) animals. **d**, All functional annotation clusters of the overlap of panel **b** (n = 110) with an enrichment score >2. **e**, Trajectories of transcription factors in untrained (light blue) and trained (blue) mKO mice that are differentially methylated after training. **f**, Bar Venn diagram of DMRs of an untrained WT or mKO muscle after an acute bout of maximal exercise (light gray or blue, respectively; 0 h and 4 h time point merged) and those of an unperturbed trained WT or mKO muscle (dark gay or blue, respectively). The common DMRs are striped (gray and blue). **g-h**, Venn diagram of WT (**g**) and mKO muscle (**h**) depicting all DMRs in untrained muscle after an acute bout of maximal exercise (open circles) and DEGs upon an acute bout of maximal exercise (colored circle). **i**, Number of DMRs in untrained sedentary mTG muscle (hypermethylated = solid bar; hypomethylated = open bar) compared to untrained sedentary WT muscle. **j**, Bar Venn diagram of DMRs of an unperturbed trained WT (dark gray) and untrained mTG (pink) muscle. The common DMRs are striped (gray and pink). **k**, Venn diagram of DMRs (open circle) and DEG (blue circle) of an untrained sedentary mTG muscle. **l**, Venn diagrams of all up- and downregulated genes in untrained sedentary mTG muscle (pink) and after an acute bout of exercise in untrained (light orange or blue) and trained (darker orange and blue) muscle. **m**, Top 3 functional annotation clusters of the up- (orange) and downregulated (blue) proteins in sedentary mTG muscle compared to sedentary WT. Data from 5 biological replicates. Data represent means ± SEM. Statistics of proteomics data were performed using empirical Bayes moderated t-statistics as implemented in the R/Bioconductor limma package and for RNA-seq data with the CLC genomics workbench software. Exact FDR-values of RNA-seq data are displayed in the Source Data file. * indicates difference to Ctrl (pre-exercise condition); *<0.05; **<0.01; ***<0.001. See also Fig. 6; Supplementary Tables 1, 2, 6, 7. Source data

In WT muscle, we have associated the transcriptomic acute exercise response of a trained muscle with epigenetic modulations of the unperturbed trained muscle (Fig. 3f–h). Therefore, we next investigated whether DNA (de-)methylation events are linked to the massive transcriptional differences in the acutely exercised, trained mKO animals. In unperturbed trained muscle, a markedly higher proportion of hypermethylated regions was found, with little overlap with DMRs in WT quadriceps that are characterized by more hypomethylation (Fig. 6f, Extended Data Fig. 8a and Supplementary Table 6). Similarly, the DEGs after acute maximal exercise associated with DMRs of trained muscle exhibited only a small overlap between the genotypes (Extended Data Fig. 8b,c). Nevertheless, many of these genes in the mKO animals partitioned to regulation of transcription, functionally similar to the results in WT animals (Extended Data Fig. 8d,e and Supplementary Table 7). Based on the largely different transcriptome of trained muscle, a divergence in DMRs might not be unexpected. However, it was surprising that absence of muscle PGC-1α also substantially altered transient epigenetic modulations in untrained muscle after an acute maximal exercise bout. In both phenotypes, little overlap exists between these transient DNA (de-)methylation events in an acute maximal exercise bout and the persistent epigenetic adaptations in unperturbed trained muscle (Extended Data Fig. 8f and Supplementary Table 6). However, although the absolute number of events after an acute maximal exercise bout at 0 h between untrained WT and mKO muscle is comparable (483 in WT, 475 in mKO), most of these DMR–gene associations are distinct (only 109 are the same). Moreover, the absolute number of DMRs in mKO muscles 4 h post-exercise is dramatically smaller than that in WT muscles (646 in WT, 80 in mKO), again with little commonality (Fig. 6g,h, Extended Data Fig. 8g,h and Supplementary Table 6). Despite all these differences between WT and mKO muscles, whenever differentially affected DMRs could be associated with corresponding genes, a strong functional cluster ‘transcription’ emerged in either phenotype, indicating that these transient DNA methylation events are closely linked to acute transcriptional regulation (Fig. 6i and Supplementary Table 7). Collectively, these results imply that PGC-1α is directly involved in the regulation of DNA methylation associated with gene expression. To further test this hypothesis, we analysed the epigenetic, transcriptomic and proteomic changes elicited in a muscle-specific PGC-1α gain-of-function model. Indeed, a substantial number of DMRs were detected in mTGs. Similar to trained WT muscle, and mirroring the outcome in mKO animals, DMRs in mTGs skewed towards hypomethylation (Extended Data Fig. 8i and Supplementary Table 6). However, the overlap between DMRs of trained WT and sedentary mTG mice was very small and only 2.8% of the transcriptionally regulated genes could be associated with DMRs (Extended Data Fig. 8j,k). In line with previous observations^31^, the transcriptome of mTGs differs substantially from the chronically and acutely training- and exercise-regulated genes in WT muscle (Extended Data Fig. 8l). A better functional representation of training adaptation is, however, provided by the mTG proteome, in which a strong accumulation of mitochondrial proteins, including members of the TCA cycle and respiratory chain, lipid metabolism and a depletion of inflammation and proteasomal catabolic processes, recapitulates many of the changes observed in trained WT muscle (Extended Data Fig. 8m and Supplementary Tables 1 and 2).

## Discussion

The plasticity evoked by exercise training leads to a pleiotropic remodelling of the function of many organs beyond muscle, with potent health benefits^1,6,7,32,33^. In light of the enormous fundamental and clinical significance of exercise, it is surprising that our understanding of the underlying processes remains incomplete. Our findings now provide evidence for a much more complex process than proposed in prevailing models, describing muscle plasticity and the corresponding basic mechanistic and regulatory principles of training adaptation.

**Fig. 7:**
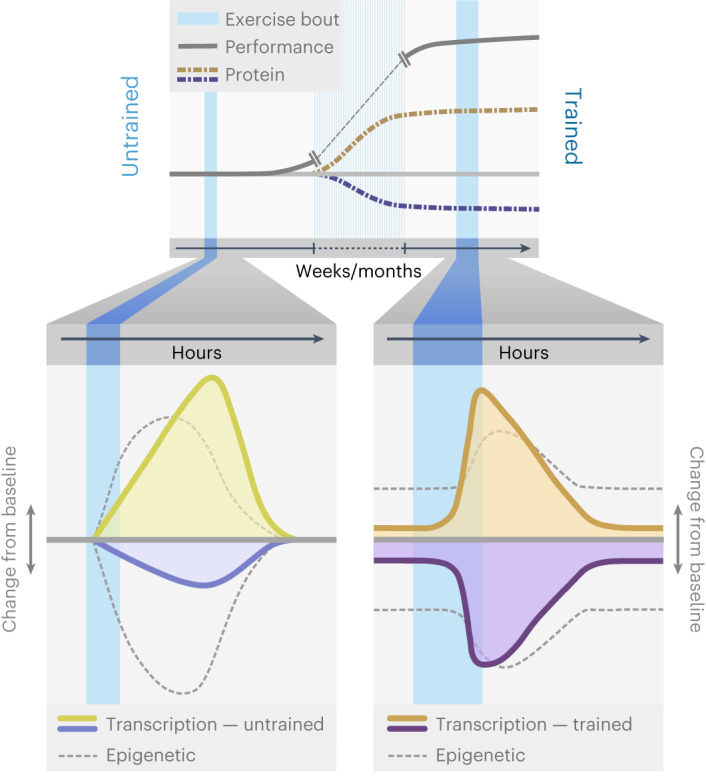
Schematic representation of the molecular exercise response. An acute bout of exercise disrupts the cellular homeostasis of the muscle and initiates a cascade of events including short-term epigenetic and transcriptional changes (change from baseline up is upregulated/hypermethylated; change from baseline down is downregulated/hypomethylated). These alterations promote the restoration of homeostasis and prepare the muscle for recurrent insults. With repeated exercise bouts over time, a trained muscle is established, hallmarked by morphological and functional adaptations that improve performance. This state is characterized by substantial proteomic remodelling, however, in the context of a small number of chronically maintained gene expression modulations. Persistent modifications of epigenetic marks prime the response of the trained muscle to recurring acute exercise bouts. Hence, a trained muscle responds more rapidly to an acute maximal exercise bout and shows a prominent repression of genes. Approximately 50% of the upregulated and 85% of the downregulated transcriptome of a trained muscle are specific to this condition and not altered in an untrained muscle post-exercise. Collectively, the molecular response to an acute bout of exercise is training status dependent and substantial qualitative and quantitative changes in gene expression events were observed in trained compared with those that occur in untrained muscle.

First, even though massive morphological and functional remodelling is necessary for training adaptation, only a small number of genes define the trained muscle transcriptionally, and steady-state gene expression changes explain only a minor subset of the corresponding modulation of the proteome. This was unexpected based on the contemporary view that repeated exercise bouts result in a persistent modulation of the basal expression of transcripts involved in mitochondrial function, substrate utilization and other functional aspects that define a trained muscle^13^. Second, the massive, yet transient remodelling of the muscle transcriptome after acute maximal exercise is quantitatively and qualitatively different when comparing an untrained with a trained muscle. Our findings vastly expand the prevailing models predicting an attenuation of the acute regulation of genes with repeated exercise bouts, in as much as we also report exacerbation, a shift in peak expression and complete disappearance and de novo emergence of numerous transcripts (Fig. 7). Finally, some transcripts exhibit a diametrically opposite expression after acute maximal exercise in untrained and trained muscles, for example, genes encoding proteins involved in ECM remodelling, inflammation or axon guidance. This suggests a training status-specific homeostatic perturbation and concomitant transcriptional response, for example, expressed in the shift from a strong stress response and damage mitigation in untrained to improved resilience in trained muscle besides metabolic, contractile and other adaptations. These highly divergent modes of adaptation imply a complex regulatory framework of training adaptation. The deconvolution analysis indicates, however, that many of these changes are mediated by events in non-muscle cells, in presumably complex multicellular crosstalk and interactions. Future studies therefore have to consider this aspect and aim for an analysis at the level of individual cell types instead of bulk muscle tissue.

Our data also shed more light on to the mechanistic underpinnings of acute maximal exercise and chronic training. We observed a clear differentiation between acute epigenetic modifications and those persistently observed in chronically trained muscle. The relatively small number of DMRs in close vicinity to differentially regulated genes in this context might be surprising, and at least in part caused by the limitation of RRBS. The association of epigenetic marks with the expression of genes modulating transcription, however, implies a priming of a limited number of key transcriptional regulators, which accordingly exhibit a different response to an acute bout of exercise in untrained and trained muscle. This priming might be sufficient for signal propagation and amplification to downstream genes, and thereby contribute to the quantitative and qualitative differences in the transcriptional networks engaged in these two settings.

From the many factors that have been implied in exercise adaptation, we investigated the regulation and function of PGC-1α. We now unequivocally demonstrate that muscle PGC-1α is indispensable for normal transcriptional muscle plasticity, both after acute maximal endurance exercise bouts in untrained and trained muscle, and in endurance-trained muscle. Moreover, we show that *V*O_2max_, a marker for maximal endurance capacity, fails to improve in mKO animals. Furthermore, training-induced shifts in the metabolism of ketone bodies and lactate are minimized in these animals^34,35^, as well as adaptations in vascularization and other processes^26,27^. Collectively, these constraints might contribute to the limited performance gains in the absence of muscle PGC-1α. Unexpectedly, we also found a strong impact of muscle PGC-1α on epigenetic marks, both chronically and acutely, and in both loss- and gain-of-function experiments. Future studies should therefore aim at investigating the molecular underpinnings of this link. Taken together, these findings demonstrate that regulatory factors such as PGC-1α, even though engaged in only an acute and transient manner, can have a profound impact on long-term training adaptations. However, the regulatory complexity of muscle plasticity might have been underestimated because redundant, alternative or contingency pathways and factors seem to be able to be recruited in such settings to re-establish adaptation. This is not only true for PGC-1α, but also for AMP-dependent protein kinase and the mammalian target of rapamycin, which are dispensable for certain aspects of training-induced muscle plasticity^36–39^. Such a complex regulatory framework would make sense in light of the evolutionary importance of the regulation of muscle plasticity, which has to function at least suboptimally to ensure survival even if individual factors fail.

Overall, our findings provide a refined and much more complex model to describe how training adaptations are brought about. These results provide insights into an unsuspected and hitherto undescribed complexity in transcriptomic, epigenetic, proteomic and phosphoproteomic changes in muscle plasticity, and hint at a vast, multifaceted mechanistic framework that controls the effects of acute exercise perturbations and long-term training alterations (Fig. 7). Once validated and expanded in both sexes, other species, age groups, muscles, training paradigms and timepoints, and in a more fine-grained cell type-specific manner, these insights will help not only to better understand such a fundamental process that is a main driver of human evolution, but also to leverage results to design strategies to benefit human health and well-being. It is encouraging that such efforts currently are ongoing, for example, in the framework of the Wu Tsai Human Performance Alliance or the Molecular Transducers of Physical Activity Consortium^40^.

## Methods

### Animals

For the present study, C57BL/6 male mice lacking PGC-1α specifically in muscle (mKO) or transgenically overexpressing PGC-1α in muscle (mTG) were used. PGC-1α mKO mice were generated by breeding PGC-1α^flox/flox^ mice with an HSA-cre mouse line (Jackson Laboratories, stock no. 009666) as described previously^41,42^. For the generation of the PGC-1α mTG animals, C57BL/6 mice expressing PGC-1α under the control of the creatine kinase promoter were crossed with WT mice as described previously^43^. Their respective littermates served as WT control animals. Mice had free access to water and a standard rodent chow diet (3432-Maintenance, KLIBA NAFAG) and were housed under standard conditions with a 12-h light:12-h dark cycle. The temperature and humidity in the animal facility were 22 ± 2 °C and 45–65%, respectively. The experiments were performed with five or six mice per condition and mice were sacrificed at the age of 18–24 weeks. We used new mouse cohorts for each analysis, except that the RRBS was performed with the same samples used for RNA-seq and proteomic and phosphoproteomic analyses were performed with the same experimental mice. All experimental protocols followed the Swiss guidelines for animal experimentation and care and were approved by the Kantonales Veterinäramt Basel-Stadt.

### Exercise protocols

Exercise training was performed on a motorized treadmill (Columbus Instruments) on 5 d per week for 1 h. The training protocol was progressive. Although the duration of the training (1 h per session) as well as the inclination of the treadmill (5°) were kept constant throughout the experiment, the speed progressively increased throughout the training period. The first training session was performed at a speed of 10 m min^−1^ and was subsequently increased by 0.5 m min^−1^ each day, resulting in a final speed of 19.5 m min^−1^ after 4 weeks. All mice were trained with one standardized training protocol, with the drawback that mKO animals trained at a higher relative intensity compared with WT mice. However, this higher relative training load of the mKO animals did not translate into a boosted adaptation. Trained mice were either sacrificed 18 h after the last training session and used as steady-state condition of a trained muscle or performed a maximal performance test after a 72-h rest period. As a control for the acute maximal exercise response of trained mice, a group of trained mice was sacrificed 72 h after the last training session, corresponding to the timepoint of the final maximal exercise bout.

Maximal oxygen consumption (*V*O_2max_) was measured during a short maximal exercise test on a closed treadmill (Columbus Instruments) with only a subset of mice. Similar to the maximal performance test, mice were first familiarized with treadmill running for 2 d. The test was performed at an inclination of 15°. After 5 min of acclimatization to the closed treadmill chamber at 0 m min^−1^, the velocity was increased to 10 m min^−1^ and then increased every 2 min by 2 m min^−1^ until exhaustion. After the test, mice were put back into their home cage.

Maximal performance and the acute exercise response to one bout of exhaustion exercise were assessed on a motorized treadmill as described previously^44^. Before the test, mice were familiarized with treadmill running for 2 d. The maximal exercise test was performed at an inclination of 5° and, after a warming up of 5 min at 5 m min^−1^ followed by 5 min at 8 m min^−1^, the velocity was progressively increased 2 m min^−1^ every 15 min until exhaustion. To determine the acute maximal exercise response, mice were sacrificed and tissue collected either immediately (0 h) post-exercise or after 4, 6 or 8 h (Fig. 2a). As maximal exercise performance varies throughout the day^45^, we decided to standardize the time of exercise and therefore perform the maximal exercise test in the morning. Hence, the dissection of the distinct timepoints was performed at different times of the day and circadian variations could not thereby be ruled out. The control group was circadian heterogeneous. After euthanizing the mice with a CO_2_ overdose, quadriceps (all four heads) of both hind limbs was removed and immediately snap-frozen in liquid nitrogen. The tissue was stored at −80 °C for further analyses. For subsequent processing, the muscle was pulverized, which allowed collection of small amounts of tissue for various analyses. The usage of pulverized muscle homogenates precludes potential fibre-type differences across specific areas of the muscle, and boosts comparability between assays. Due to technical limitations, the tissue amount was limiting and we were therefore unable to use the muscle from the same experimental mice for all analyses. Hence, proteomic and phosphoproteomic analyses were performed with one and RNA-seq and RRBS with a second cohort of experimental mice.

The term ‘acute exercise’ is used to describe the acute perturbation that occurs with one bout of exercise (regardless of whether the mice were untrained or trained). In contrast, ‘training’ is the result of repeating the exercise for a prolonged period of time, eventually leading to chronic training adaptations. Steady-state changes in a trained muscle are investigated without an acute bout of exercise before the dissection (‘unperturbed muscle’).

### RNA-seq and data analysis

After homogenizing pulverized quadriceps in 1 ml of TRIzol agent (Sigma-Aldrich) using FastPrep tubes (MP Biomedicals), RNA was isolated according to the manufacturer’s protocol. RNA concentration and quality were measured on the NanoDrop OneC spectrophotometer (Thermo Fisher Scientific). Subsequently, 7,500 ng of RNA was further purified using the Direct-zol RNA MiniPrep Kit (Zymo Research). After the RNA-seq library preparation with 1 µg of purified RNA using the TruSeq RNA library Prep Kit (Illumina) according to the manufacturer’s instructions, single-read sequencing was performed on the HiSeq 2500 machine (51 cycles, Illumina).

**Extended Data Fig. 9:**
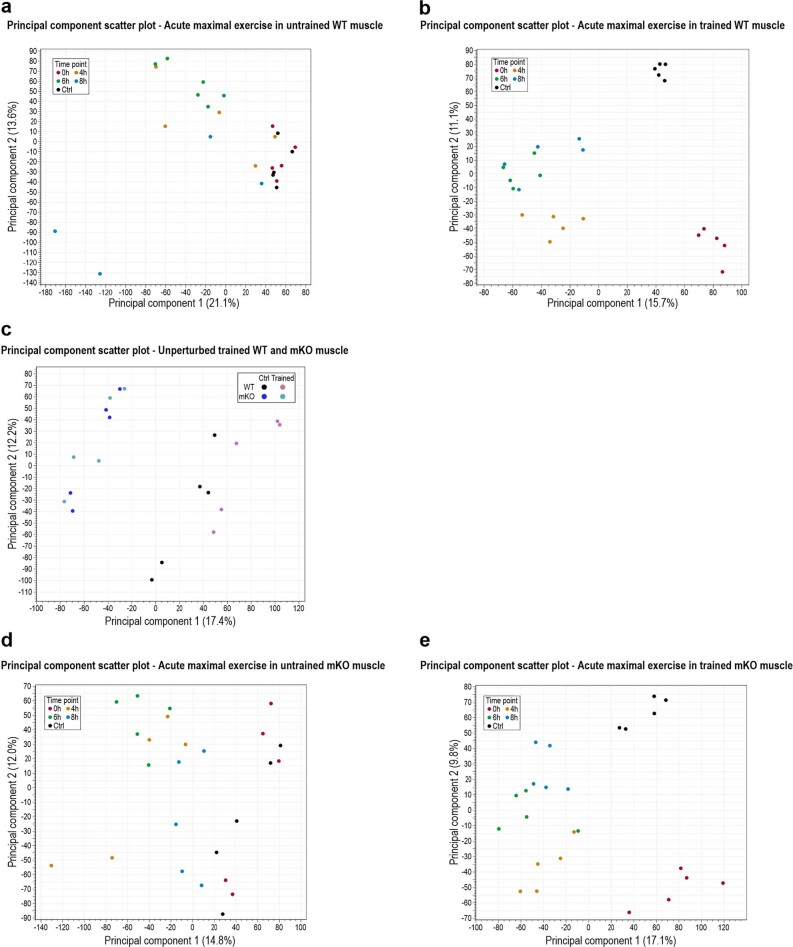
PCA scatter plot of the different RNA-seq datasets. **a, b**, Principal component analysis (PCA) scatter plots of RNA-seq data of untrained (**a**) and trained (**b**) WT muscle after an acute bout of maximal exercise. **c**, PCA scatter plot of RNA-seq data of untrained and trained unperturbed WT and mKO muscle. **d, e**, PCA scatter plots of RNA-seq data of untrained (**d**) and trained (**e**) mKO muscle after an acute bout of maximal exercise.

All RNA-seq analyses were performed using the CLC Genomics Workbench Software (v.21.0.5, QIAGEN). Before mapping the reads to the mm10 version of the mouse genome, reads were quality and adaptor trimmed. For the differential gene expression analyses, the TMM (trimmed mean of M-values) method was used for normalization. Principal component analysis (PCA) scatter plots were used to visualize the sample distribution (Extended Data Fig. 9). All steps including quality control and differential gene expression analyses were performed using the CLC Genomics Workbench Software. To visualize gene expression, the relative expression was calculated from the transcripts per million (TPM) provided by the software. For all downstream analysis, a log_2_(fold-change) (log_2_(FC)) cut-off of ±0.6 (genes with a log_2_(FC) >0.599 or <−0.599) and a false discovery rate (FDR) value <0.05 were used. The prediction of enriched transcription factor-binding motifs was done by ISMARA (https://ismara.unibas.ch/mara)^21^. The Database for Annotation, Visualization and Integrated Discovery (DAVID; https://david.ncifcrf.gov/tools.jsp) platform was used to determine functional annotation clusters of gene ontology (GO) biological processes and REACTOME pathways and clusters with an enrichment score >2 were considered^46,47^. The overlap of genes was determined using InteractiVenn (http://www.interactivenn.net/index.html)^48^ and results were visualized with dot plots or heatmaps using Morpheus (https://clue.io/morpheus) or proportional Venn diagrams using DeepVenn (https://www.deepvenn.com)^49^.

### Genomic DNA isolation

Approximately 15 mg of pulverized quadriceps was used for genomic DNA (gDNA) isolation. Tissue was digested overnight in proteinase K (20 mg ml^−1^) (Promega) and DNA lysis buffer (50 mM Tris-HCl, pH 8.0, 100 mM NaCl, 10 mM EDTA and 0.5% Nonidet P-40) at 55 °C on a shaker. The next day proteinase K was inactivated at 95 °C for 10 min. Subsequently, phenol–chloroform–isoamyl alcohol (PCI) (Sigma-Aldrich) was added in a 1:1 ratio and the samples were vortexed and centrifuged at room temperature (RT) and 16,000*g* for 4 min. Next, the upper phase was collected, and the same volume of PCI as in the first step added, vortexed and centrifuged as described above. Then, the upper phase was collected again and 1:10 volume of 3 M Na acetate, pH 5.0 and 6:10 volume of isopropanol were added. The samples were vortexed, incubated at RT for 5 min and centrifuged at RT at maximum speed for 15 min. Subsequently, the supernatant was removed and the pellet washed with 70% ethanol and centrifuged at RT at maximum speed for 5 min. After removing the supernatant, the pellet was dried for 10 min at RT and resuspended in nuclease-free H_2_O. The gDNA quality and concentration were measured on the NanoDrop OneC spectrophotometer (Thermo Fisher Scientific). The isolated gDNA was further purified according to the manufacturer’s instructions using the DNeasy Blood & Tissue Kit (QIAGEN) and quality and concentration measured on the NanoDrop OneC spectrophotometer (Thermo Fisher Scientific).

### RRBS and identification of DMRs

The RRBS library was prepared with the Premium RRBS Kit (Diagenode) according to the manufacturer’s instructions with 100 ng of gDNA as starting material. Quality and fragment size were determined with the Bioanalyzer (Agilent). Single-read sequencing was performed with a HiSeq2500 machine (51 cycles, Illumina).

The reads were quality and adaptor trimmed with the Trim Galore! (v.0.4.5) wrapper of cutadapt^50^. The trimmed reads were controlled with FastQC (http://www.bioinformatics.bbsrc.ac.uk/projects/fastqc). Conversion rates were calculated with customized scripts, counting the number of Gs and Cs in non-GC context, resulting in values >99% for all libraries. The reads were mapped to the mm10 version of the mouse genome with BWA^51^ and methylCtools^52^ after a slightly extended bis-SNP pipeline^53^. The reads were locally realigned and the quality values were recalibrated before calling the methylation levels. The mm10 SNPs and indels from dbSNP v.138 were used in this process^54^. An initial quality control and exploratory analysis were done with R package RnBeads^55^. Differential loci were detected with MethylKit^56^ testing in 500-bp sliding windows with at least three CpGs, including only those with a coverage of at least 10×. DMRs were defined as ±10% with a *q*-value < 0.01.

### Proteomics and phosphoproteomics

#### Sample preparation

Approximately 10 mg of pulverized quadriceps was used for sample preparation. The muscles of mTG mice and WT littermates (referred to as group A) were lysed in 8 M urea, 0.1 M ammonium bicarbonate and phosphatase inhibitors (Sigma-Aldrich) by sonication (Bioruptor, 10 cycles, 30 s on/off, Diagenode) and proteins were digested as described previously^45,57^. The muscles of mKO and WT littermate mice were resuspended in lysis buffer containing 5% sodium dodecylsulfate, 10 mM tris(2-carboxyethyl)phosphine (TCEP) and 0.1 M tetraethylammonium bromide, and lysed by sonication using a PIXUL Multi-Sample Sonicator (Active Motif) with the pulse set to 50, pulse repetition frequency to 1, process time to 20 min and burst rate to 20 Hz. Lysates were incubated for 10 min at 95 °C, alkylated in 20 mM iodoacetamide for 30 min at 25 °C and proteins either digested using S-Trap micro-spin columns (Protifi), according to the manufacturer’s instructions (referred to as group B), or further processed for phosphoproteomic analysis (referred to as group C). Samples of group C were trichloroacetic acid precipitated according to a protocol originally from Luis Sanchez (https://www.its.caltech.edu/~bjorker/TCA_ppt_protocol.pdf). Pellets were resuspended in 2 M guanidinium-HCl, 0.1 M ammonium bicarbonate, 5 mM TCEP and phosphatase inhibitors (Sigma-Aldrich, catalogue no. P5726&P0044) and proteins were digested as described previously^45,57^. Enrichment for phosphorylated peptides (group C) was performed using Fe[III]-IMAC cartridges on an AssayMAP Bravo platform following a recently described method^58^.

Dried peptides (of group A and B) as well as the phospho-enriched peptides (group C) were resuspended in 0.1% aqueous formic acid and subjected to liquid chromatography–tandem mass spectrometry (LC/MS–MS) analysis using a Orbitrap Fusion Lumos Mass Spectrometer fitted with an EASY-nLC 1200 (both Thermo Fisher Scientific) and a customized column heater set to 60 °C as described previously^45^.

For samples of group A and C, the mass spectrometer was operated in data-dependent acquisition mode with a cycle time of 3 s between master scans as described for phosphoproteomic samples previously^45^. For the proteomic samples of group A, changes have been made: the master scan was acquired at a resolution of 240,000 full width at half-maximum (FWHM; at 200 *m*/*z*) and was followed by MS2 scans of the most intense precursors in the linear ion trap at ‘rapid’ scan rate. Furthermore, maximum ion injection time for MS2 was set to 35 ms. Finally, the intensity threshold was set to 5,000 and collision energy to 35%.

For samples of group B, the mass spectrometer was operated in data-independent acquisition (DIA) mode. The MS1 scans were obtained using the Orbitrap in centroid mode with a resolution of 120,000 FWHM (at 200 *m*/*z*). The scan range was from 390 to 1,210 *m*/*z*, the automatic gain control (AGC) target set to 800% and a maximum ion injection time of 100 ms. The MS2 scans was acquired at a resolution of 15,000 FWHM (at 200 *m*/*z*) in the Orbitrap in centroid mode. The precursor mass range was set from 400 to 1,200, and a quadrupole isolation window of 8 *m*/*z* with a 1*-m*/*z* window overlap was used. The scan range for MS2 was from 145 *m*/*z* to 1,450 m/z, the AGC target was set to standard and the maximum ion injection time was 22 ms. Higher-energy collisional dissociation was employed for peptide fragmentation and the collision energy was set to 33%. One microscan was acquired for each spectrum.

#### Data analysis

The raw files obtained from samples of group A and C were imported into the Progenesis QI software (v.2.0, Nonlinear Dynamics Limited). This software was utilized with default parameters to extract peptide precursor ion intensities across all samples. The generated mgf-files were searched using MASCOT against a murine database (consisting of 17,013 Swiss-Prot protein sequences downloaded from Uniprot on 20190307 for group A and 34,186 forward and reverse protein sequences were downloaded from Uniprot on 20220222 for group C, https://www.uniprot.org/taxonomy/10090) and 392 commonly observed contaminants using the search criteria described previously for the phosphoproteomics data^45^. Two modifications were made for group A: instead of phosphorylation (STY) as a variable modification, acetyl (protein amino terminal) was used. Furthermore, mass tolerance of 10 p.p.m. (precursor) and 0.6 Da (fragments) was considered.

For group B, the acquired raw files were searched using the Spectronaut (Biognosys v.15.7) directDIA workflow against a murine database (consisting of 17,093 Swiss-Prot protein sequences downloaded from Uniprot on 20220222, https://www.uniprot.org/taxonomy/10090) and 392 commonly observed contaminants. The default factory settings were employed with slight adjustments. Specifically, in the Pulsar Search Result Filter tab, the fragment ion *m*/*z* range was set to 300–1,800 and the relative intensity minimum to 5.

Quantitative analysis results from label-free quantification or exported from Spectronaut were processed using the SafeQuant R package v.2.3.2 (https://github.com/eahrne/SafeQuant)^57^ to obtain peptide relative abundances. In this analysis, global data normalization was conducted by equalizing the total peak/reporter areas across all LC/MS–MS runs. Data imputation was performed using the k-nearest neighbours algorithm to handle missing values. Subsequently, peak areas were summed per protein and LC/MS–MS run and peptide abundance ratios were calculated. Only isoform-specific peptide ion signals were considered for quantification. To fulfil additional assumptions such as normality and homoscedasticity required for the application of linear regression models and Student’s *t*-tests, the MS-intensity signals were transformed from the linear scale to the log(scale). The summarized peptide expression values were then used to statistically test the differential abundance of peptides between the conditions. In this context, empirical Bayes-moderated *t*-statistics tests were applied, as implemented in the R/Bioconductor limma package (http://bioconductor.org/packages/release/bioc/html/limma.html). Three proteomic samples failed quality control and had to be excluded from the analysis (one WT sedentary, one WT-trained and one mKO-trained). These were also excluded from the phosphoproteomic analysis. For all proteomic analyses, only proteins with more than one peptide were considered. In addition, a log_2_(FC) cut-off of ±0.2 (proteins with a log_2_(FC) >0.199 or <−0.199) was used for all analyses and a *P* value <0.05 was considered statistically significant.

### ScRNA-seq and snRNA-seq data analysis

To create the single-transcriptomic reference dataset including both mononucleated cells and myonuclei, we integrated published single-cell data (scRNA-seq) from mononucleated muscle cells^23^ and single-nucleus data (snRNA-seq) from myonuclei^22^. In detail, we subsetted the provided scRNA-seq data from ref. ^23^ for all samples from skeletal muscle and reanalysed it via R/Seurat 4.0, including NormalizeData(), FindVariableFeatures() (with the top 3,000 variable genes), ScaleData() (regressing out mitochondrial genes) and PCA^23^. Then, we integrated the samples using Harmony (github.com/immunogenomics/harmony) and applied clustering via FindNeighbors(), FindClusters() and RunUMAP(). Finally, we annotated clusters based on published marker gene expression and removed cells from high-fat-diet-fed mice. To complement the scRNA-seq data from mononucleated muscle cells with missing myonuclei data, we used published snRNA-seq from the tibialis anterior muscles^22^. In short, we applied quality measures as described in the original publication and clustered nuclei with the common Seurat v.4.0 pipeline. After cluster annotation, we subsetted the dataset for myonuclei only. Subsequent integration of scRNA-seq and snRNA-seq data was performed by merging all datasets and recalculating the normalization, variable features, scaling and principal components. We corrected for batch effects and integrated the individual samples via Harmony and clustered as described above. To visualize the expression of a given set of genes, we used the Clustered_DotPlot() function of the ‘scCustomize’ package (samuel-marsh.github.io/scCustomize) with the minimum colour threshold set to zero.

### Statistical analysis

The statistical analyses of the RNA-seq, RRBS and proteomic analyses were done as described in the respective sections. All other statistical analyses were performed in GraphPad Prism v.9 using two-tailed Student’s *t*-test or two-way analysis of variance (ANOVA) followed by Šídák’s multiple-comparison test. Values are expressed as mean ± s.e.m. except for data presented in box plots that display the median and the 25th to 75th percentiles and whiskers indicating the minimal and maximal values. Generally, *P* < 0.05 was considered statistically significant. As an exception, FDR < 0.05 for RNA-seq analysis, *q*-value < 0.01 for RRBS analysis and *z*-score > 1.96 for ISMARA were considered statistically significant.

### Reporting summary

Further information on research design is available in the Nature Portfolio Reporting Summary linked to this article.

### Supplementary information

Reporting Summary

Supplementary Tables **Supplementary Table 1** Significantly changed proteome (cut-off: peptide >1; *P* < 0.05; log_2_(FC) ± 0.2) in quadriceps of sedentary and trained WT and mKO animals, as well as sedentary mTG mice and phosphoproteome (cut-off: *P* < 0.05; log_2_(FC) ± 0.2) in quadriceps of sedentary and trained WT and mKO animals. **Supplementary Table 2** Functional annotation clusters of GO biological processes and REACTOME pathways with an enrichment score >2 using DAVID of significantly changed proteome of trained WT and mKO mice as well as sedentary mKO and mTG animals and functional annotation of GO biological processes with FDR < 0.05 using DAVID of significantly changed phosphoproteome of trained WT and mKO mice. **Supplementary Table 3** Functional annotation clusters of GO biological processes and REACTOME pathways with an enrichment score >2 using DAVID of DEGs in WT mice in response to an acute bout of exercise or after training. **Supplementary Table 4** All predicted motifs with significantly changed activities using ISMARA of WT and mKO animals in response to an acute bout of exercise or after training. **Supplementary Table 5** Functional annotation clusters of GO biological processes and REACTOME pathways with an enrichment score >2 using DAVID of proteins that are transcriptionally regulated in either trained WT muscle or after an acute exercise bout in WT animals. **Supplementary Table 6** Differentially methylated regions (defined as ±10% with a *q*-value < 0.01) in quadriceps of sedentary and trained WT and mKO animals, untrained WT and mKO animals 0 and 4 h post-exercise, as well as sedentary mTG mice. **Supplementary Table 7** Functional annotation clusters of GO biological processes and REACTOME pathways with an enrichment score >2 using DAVID of DEGs with differentially methylated regions of sedentary and trained WT and mKO mice, as well as sedentary mTG animals. **Supplementary Table 8** Functional annotation clusters of GO biological processes and REACTOME pathways with an enrichment score >2 using DAVID of DEGs in mKO mice in response to an acute bout of exercise or after training, as well as those that are exclusively regulated in WT muscle.

### Source data

Source Data Figs. and Extended Data Figs Data and statistical source data for Figs. 1–6 and Extended Data Figs. 1–8.

## Data Availability

Transcriptomic and RRBS data have been deposited at the Gene Expression Omnibus (accession nos. GSE221210 and GSE221831, respectively). The transcriptomic data are furthermore accessible in an analysed form at Myo-TrEx (https://myo-trex.scicore.unibas.ch). Proteomic data have been deposited at the proteomics identifications database (MassIVE, accession no. MSV000092203 and ProteomeXchange, accession no. PXD043097). Source data are provided with the present paper.

## Code availability

Data have been analysed using either commercial tools (that is, CLC Genomics Workbench Software) or existing standard packages and scripts described in Methods. No new code has been developed.
