# Supplementary material for: Molecular control of endurance training adaptation in male mouse skeletal muscle
Source: Nat Metab. 2023 Sep 11;5(11):2020–35. doi: 10.1038/s42255-023-00891-y (PMC10663156; doi:10.1038/s42255-023-00891-y)
Supplement: Supplementary file 1 — Reporting Summary [file 42255_2023_891_MOESM1_ESM.pdf]

nature portfolio

Corresponding author(s): Christoph Handschin

Last updated by author(s): Aug 3, 2023

Reporting Summary

Nature Portfolio wishes to improve the reproducibility of the work that we publish. This form provides structure for consistency and transparency in reporting. For further information on Nature Portfolio policies, see our Editorial Policies and the Editorial Policy Checklist.

Statistics

For all statistical analyses, confirm that the following items are present in the figure legend, table legend, main text, or Methods section.

n/a	Confirmed	
☐	☒	The exact sample size (*n*) for each experimental group/condition, given as a discrete number and unit of measurement
☐	☒	A statement on whether measurements were taken from distinct samples or whether the same sample was measured repeatedly
☐	☒	The statistical test(s) used AND whether they are one- or two-sided<br>*Only common tests should be described solely by name; describe more complex techniques in the Methods section.*
☒	☐	A description of all covariates tested
☐	☒	A description of any assumptions or corrections, such as tests of normality and adjustment for multiple comparisons
☐	☒	A full description of the statistical parameters including central tendency (e.g. means) or other basic estimates (e.g. regression coefficient) AND variation (e.g. standard deviation) or associated estimates of uncertainty (e.g. confidence intervals)
☐	☒	For null hypothesis testing, the test statistic (e.g. *F*, *t*, *r*) with confidence intervals, effect sizes, degrees of freedom and *P* value noted<br>*Give P values as exact values whenever suitable.*
☐	☒	For Bayesian analysis, information on the choice of priors and Markov chain Monte Carlo settings
☒	☐	For hierarchical and complex designs, identification of the appropriate level for tests and full reporting of outcomes
☒	☐	Estimates of effect sizes (e.g. Cohen's *d*, Pearson's *r*), indicating how they were calculated

Our web collection on statistics for biologists contains articles on many of the points above.

Software and code

Policy information about availability of computer code

Data collection	RNA-seq and RRBS were performed on the Illumina HiSeq 2500.<br>Proteomics and phosphoproteomics data was collected on the Thermo EASY-nlC Orbitrap Fusion Lumos.
Data analysis	All sequencing data were mapped to the mm10 version of the mouse genome.<br>RNA-seq analysis was performed using the the CLC Genomics Workbench Software (version 21.0.5, Qiagen).<br>RRBS analysis: The reads were quality- and adapter-trimmed with the Trim Galore! (Version 0.4.5) wrapper of cutadapt. The trimmed reads were controlled with FastQC (http://www.bioinformatics.bbsrc.ac.uk/projects/fastqc/). Conversion rates were calculated with custom scripts, counting the amount of G's and C's in non-GC context resulting in values above 99% for all libraries. The reads were mapped to the mm10 version of the mouse genome with BWA and methylCtools after a slightly extended Bis-SNP pipeline. The reads were locally realigned and the quality values were recalibrated before calling the methylation levels. The mm10 SNPs and InDels from dbSNP v138 was used in this process. An initial quality control and exploratory analysis was done with R package RnBeads. Differential loci were detected with MethylKit testing in 500 bp sliding windows with at least 3 CpGs, only including those with a coverage of at least 10x.<br>Proteomics and phosphoproteomics analyses were performed using the Spectronaut (Biognosys v15.7) direct DIA workflow and Progenesis QI software (v2.0, Nonlinear Dynamics Limited). Quantitative data was exported fand analyzed using the SafeQuant R package v.2.3.2. (https://github.com/eahrne/SafeQuant/). This analysis included data imputation using the knn algorithm, summation of peak areas per protein and LC-MS/MS run, followed by calculation of protein/peptide abundance ratios and testing for differential abundance using empirical Bayes moderated t-statistics (as implemented in the R/Bioconductor limma package).<br>Single cell and single nuclei RNA-seq data analysis was performed using R/Seurat4.0 including following functions: NormalizeData(), FindVariableFeatures(), ScaleData(), FindNeighbors(), FindClusters(), and RunUMAP(), and the implemented function RunHarmony() of the Harmony package (github.com/immunogenomics/harmony).

All other statistical analyses were performed in GraphPad Prism 9.

For manuscripts utilizing custom algorithms or software that are central to the research but not yet described in published literature, software must be made available to editors and reviewers. We strongly encourage code deposition in a community repository (e.g. GitHub). See the Nature Portfolio guidelines for submitting code & software for further information.

Data

Policy information about availability of data

All manuscripts must include a data availability statement. This statement should provide the following information, where applicable:

- Accession codes, unique identifiers, or web links for publicly available datasets
- A description of any restrictions on data availability
- For clinical datasets or third party data, please ensure that the statement adheres to our policy

Transcriptomic and RRBS data have been deposited at the Gene Expression Omnibus (GEO, accession numbers GSE221210 and GSE221831, respectively). The transcriptomic data are furthermore accessible in an analyzed form at the myo-transcriptome of exercise database (myoTrEx, https://myo-trex.scicore.unibas.ch). Proteomic and phosphoproteomic data have been deposited at the Proteomics Identifications Database (MassIVE, accession number MSV000092203).

Human research participants

Policy information about studies involving human research participants and Sex and Gender in Research.

Reporting on sex and gender	Not applicable. No human participants were used in this study.
Population characteristics	Not applicable. No human participants were used in this study.
Recruitment	Not applicable. No human participants were used in this study.
Ethics oversight	Not applicable. No human participants were used in this study.

Note that full information on the approval of the study protocol must also be provided in the manuscript.

Field-specific reporting

Please select the one below that is the best fit for your research. If you are not sure, read the appropriate sections before making your selection.

☒ Life sciences ☐ Behavioural & social sciences ☐ Ecological, evolutionary & environmental sciences

For a reference copy of the document with all sections, see nature.com/documents/nr-reporting-summary-flat.pdf

Life sciences study design

All studies must disclose on these points even when the disclosure is negative.

Sample size	The sample size is indicated in the figure or figure legend. No sample size calculation was performed but the sample size was based on our experience with these kind of interventions and measurement (PMID: 34142717, 24277823) as well as the common practice in the field (DOI: 10.1101/2022.09.21.508770). Complying to the 3R rule, we tried to use a limited number of animals that still allowed to reach statistical robust and meaningful results.
Data exclusions	Three samples had to be excluded from the proteomics analysis (one WT-sedentary, one WT-trained and one mKO-trained). These mice showed abnormal clustering. These criteria were defined prior to the analysis.
Replication	We had at least 5 biological replicates per cohort and analysis. Since we performed the same intervention (acute exercise and/or training) with a lot of different groups (that were either sacrificed at different time points or used for distinct analysis), we replicated the effect of the intervention on performance in both genotypes multiple times successfully.
Randomization	Mice were genotype-matched and randomly assigned to one of the intervention groups (i.e., sedentary, acute exercise, training or training +acute exercise).
Blinding	The researchers were not blinded to the group allocation of the mice since they performed the training intervention. However, the researchers were blinded for the genotype of the mice.

Reporting for specific materials, systems and methods

We require information from authors about some types of materials, experimental systems and methods used in many studies. Here, indicate whether each material, system or method listed is relevant to your study. If you are not sure if a list item applies to your research, read the appropriate section before selecting a response.

Materials & experimental systems

n/a	Involved in the study
☒	☐ Antibodies
☒	☐ Eukaryotic cell lines
☒	☐ Palaeontology and archaeology
☐	☒ Animals and other organisms
☒	☐ Clinical data
☒	☐ Dual use research of concern

Methods

n/a	Involved in the study
☒	☐ ChIP-seq
☒	☐ Flow cytometry
☒	☐ MRI-based neuroimaging

Animals and other research organisms

Policy information about studies involving animals; ARRIVE guidelines recommended for reporting animal research, and Sex and Gender in Research

Laboratory animals	All mice had a C57BL/6 background. PGC-1α mKO mice were generated by breeding PGC-1αflox/flox mice with a HSA-cre mouse line (Jackson Laboratories stock number: 009666) as previously described (PMID: 15454086, 17932564). For the generation of the PGC-1α mTG animals, C57BL/6 mice expressing PGC-1α under the control of the creatine kinase promoter were crossed with WT mice as described previously (PMID: 12181572). Mice were at the age of 18-24 weeks.<br>Mice had free access to water and a standard rodent chow diet (3432-Maintenance, KLIBA NAFAG) and were housed under standard conditions with a 12h light/12h dark cycle. The temperature and humidity in the animal facility ranged between 22 ± 2°C and 45-65%, respectively.
Wild animals	No wild animals were used for this study.
Reporting on sex	The findings presented in this study only apply to male mice and have not been tested in female mice.
Field-collected samples	This study does not contain field-collected samples.
Ethics oversight	All experimental protocols followed the Swiss guidelines for animal experimentation and care and were approved by the Kantonales Veterinäramt Basel-Stadt.

Note that full information on the approval of the study protocol must also be provided in the manuscript.
